# A Global Map of G Protein Signaling Regulation by RGS Proteins

**DOI:** 10.1016/j.cell.2020.08.052

**Published:** 2020-10-15

**Authors:** Ikuo Masuho, Santhanam Balaji, Brian S. Muntean, Nickolas K. Skamangas, Sreenivas Chavali, John J.G. Tesmer, M. Madan Babu, Kirill A. Martemyanov

**Affiliations:** 1 Department of Neuroscience, The Scripps Research Institute Florida, Jupiter, FL 33458, USA; 2 MRC Laboratory of Molecular Biology, Francis Crick Avenue, Cambridge CB2 0QH, UK; 3 Departments of Structural Biology and Center for Data Driven Discovery, St. Jude Children’s Research Hospital, Memphis, TN 38105, USA; 4 Department of Biology, Indian Institute of Science Education and Research (IISER) Tirupati, Karakambadi Road, Tirupati 517 507, India; 5 Departments of Biological Sciences and Medicinal Chemistry and Molecular Pharmacology, Purdue University, West Lafayette, IN 47907-2054, USA

**Keywords:** RGS, G protein, GPCR, BRET, genetic variation, ancestral reconstitution, protein-protein interaction, cell signaling, striatum

## Abstract

The control over the extent and timing of G protein signaling is provided by the regulator of G protein signaling (RGS) proteins that deactivate G protein α subunits (Gα). Mammalian genomes encode 20 canonical RGS and 16 Gα genes with key roles in physiology and disease. To understand the principles governing the selectivity of Gα regulation by RGS, we examine the catalytic activity of all canonical human RGS proteins and their selectivity for a complete set of Gα substrates using real-time kinetic measurements in living cells. The data reveal rules governing RGS-Gα recognition, the structural basis of its selectivity, and provide principles for engineering RGS proteins with defined selectivity. The study also explores the evolution of RGS-Gα selectivity through ancestral reconstruction and demonstrates how naturally occurring non-synonymous variants in RGS alter signaling. These results provide a blueprint for decoding signaling selectivity and advance our understanding of molecular recognition principles.

## Highlights

• Systematic analysis reveals G protein selectivity of all canonical RGS proteins
• RGS proteins rely on selectivity bar codes for selective G protein recognition
• Transplantation of bar codes across RGS proteins switches their G protein preferences
• Natural variants, mutations, and evolution shape RGS selectivity

## Graphical Abstract

**Figure:**
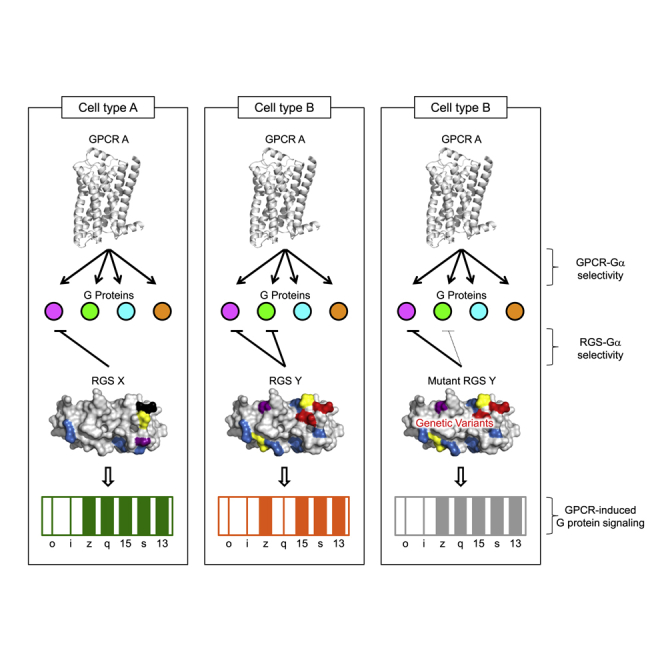

## Teaser

Masuho et al. quantitatively define G protein selectivity of all of the canonical members of the regulator of G protein signaling (RGS) family. Based on this information, they determine the structural basis of selective RGS-G protein recognition and demonstrate how selectivity determinants evolved. They further show that the RGS-G protein selectivity is affected by the mutational genomic landscape and can be rationally altered.

## Introduction

Heterotrimeric G proteins transduce a vast variety of extracellular stimuli, including hormones, ions, organic molecules, and light into the regulation of intracellular “effectors” to generate cellular responses (Neves et al., 2002). Collectively, G protein systems play a role in nearly every physiological process and in numerous pathologies (Heng et al., 2013; Kostenis et al., 2020; O’Hayre et al., 2014; Wang et al., 2018). G proteins are activated by the binding of GTP to the α subunits (Gα) that release them from inhibitory occlusion by the βγ dimer (Gβγ) (Glukhova et al., 2018; Lambert, 2008; Oldham et al., 2006; Syrovatkina et al., 2016). Mammalian genomes encode a conserved set of 16 Gα subunits, each possessing unique signaling properties and the ability to selectively engage a distinct set of effectors, including adenylate cyclases, phospholipase C isozymes, Rho guanine nucleotide exchange factors (GEFs), and ion channels (Hubbard and Hepler, 2006; Marinissen and Gutkind, 2001; Wettschureck and Offermanns, 2005).

The key determinant of G protein action in cells is their lifetime in an active state. Thus, the activation and deactivation of G proteins is tightly controlled and ought to occur with selectivity for individual G proteins to ensure the selectivity of downstream signaling (Siderovski and Willard, 2005; Syrovatkina et al., 2016; Wettschureck and Offermanns, 2005). Deciphering molecular mechanisms of this selectivity is of paramount importance for understanding how the signals are routed in the cells. A number of G protein activators have been described and demonstrated to act as GEFs on the Gα subunits with clear subtype selectivity (Cismowski et al., 1999; Garcia-Marcos et al., 2011; Tall et al., 2003). Among them, the largest class is the G protein-coupled receptor (GPCR) family (Fredriksson et al., 2003; Hilger et al., 2018; Mahoney and Sunahara, 2016). GPCRs exhibit clear preferences for activating particular Gα species, and there has been tremendous progress in understanding the molecular mechanisms in establishing this selectivity (Flock et al., 2017; Inoue et al., 2019; Masuho et al., 2015b; Okashah et al., 2019).

The opposing process of G protein deactivation occurs when G proteins hydrolyze guanosine triphosphate (GTP), a process assisted by the action of the GTPase-activating proteins (GAPs). The GAP action is essential for avoiding response saturation and for achieving temporal resolution dictated by individual physiological reactions (Ross, 2008). Most well-characterized GAPs for heterotrimeric G proteins belong to the regulator of G protein signaling (RGS) family, consisting of 20 canonical members in mammals (Dohlman and Thorner, 1997; Tesmer, 2009). RGS proteins bind to active Gα proteins and facilitate their GTPase activity, thereby accelerating the termination of G protein signaling (Berman et al., 1996b; Hunt et al., 1996; Ross and Wilkie, 2000; Saitoh et al., 1997; Watson et al., 1996). It is now well established that this action of RGS proteins is crucial for achieving the physiologically relevant timing and extent of GPCR signaling (Hollinger and Hepler, 2002; Kimple et al., 2011; Neubig, 2015). Accordingly, the loss of RGS-mediated control leads to a range of pathologies observed in mouse models (Bansal et al., 2007; Gaspari et al., 2018; Lee et al., 2010; Senese et al., 2020) and is increasingly associated with human diseases (Shamseldin et al., 2016; Squires et al., 2018). Studies in several members of the RGS family indicate that they exert considerable selectivity in recognizing Gα (Heximer et al., 1997; Snow et al., 1998; Soundararajan et al., 2008; Tesmer, 2009; Wang et al., 1998). There has been significant progress documenting cases of selective RGS-Gα interactions (Hollinger and Hepler, 2002), analyzing the structural basis for this selectivity (Soundararajan et al., 2008; Taylor et al., 2016), and mapping amino acid residues involved in specific recognition (Kimple et al., 2009; Kosloff et al., 2011). Although these studies provide insights into the selectivity of RGS action for isolated cases, a comprehensive understanding of the complete landscape of Gα preferences of RGS proteins is still lacking.

This study presents a map of Gα selectivity for all canonical RGS proteins. We monitored the temporal regulation of GPCR-mediated G protein signaling and quantitatively characterized the GAP activity of the RGS proteins, testing nearly all of the theoretically possible Gα-RGS pairings (300 combinations). Using the functional activity as a readout in the context of a physiologically relevant cellular environment allowed us ot document the preferences of RGS proteins for Gα substrates, revealing pairings and disallowed combinations. This information led to the identification of molecular determinants involved in the selectivity of Gα-RGS recognition. Applying computational algorithms, we also show how these determinants have evolved and can be used to create designer RGS proteins with novel selectivity profiles. Analysis of human genomic data further suggests that genetic variations in RGS selectivity determinants may contribute to non-disease traits, pathological dysregulation of GPCR signaling, and variable responsiveness to drug treatments.

## Results

### Assaying Activity of All Canonical RGS Proteins on Gα Deactivation with a Real-Time Kinetic Approach in Living Cells

**Figure 1:**
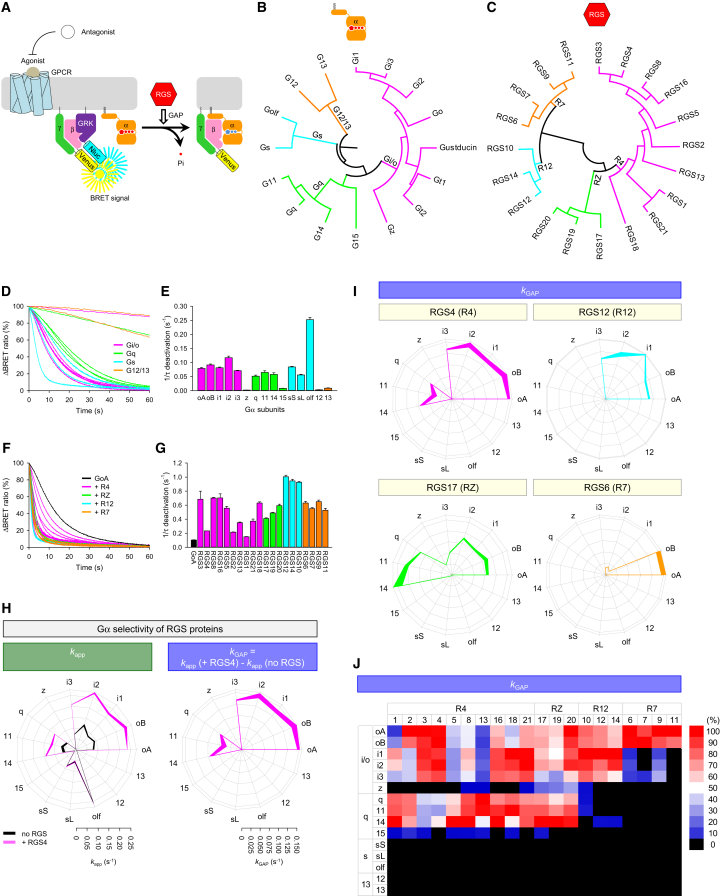
Determining Gα Selectivity of All Canonical RGS Proteins in Living Cells (A) Schematic of the BRET assay. Agonist-bound GPCR leads to the dissociation of inactive heterotrimeric G proteins into active GTP-bound Gα and Venus-Gβγ subunits. The free Venus-Gβγ interacts with the Gβγ-effector mimetic masGRK3ct-Nluc-HA and increases the BRET signal. The application of the antagonist initiates the deactivation of G proteins and decreases the BRET signal. (B and C) Phylogenetic trees of Gα subunits and RGS proteins. (D and E) The deactivation time course of 15 different G proteins. (F and G) The effect of RGS proteins on the deactivation of Gα_oA_. (H) Quantification of RGS action in G protein regulation. The rate constants in the absence (black) and presence of RGS4 (pink, left), and subtracted *k*_GAP_ value for RGS4 (pink, right) are shown. (I) Gα selectivity fingerprints for representative RGS proteins. The *k*_GAP_ were normalized to the largest value and plotted as corresponding vertices. The thickness of the lines represents the SEM of 3 independent experiments. Linear scale is used. (J) Heatmap of the normalized *k*_GAP_ values. The black “0” values are assigned when no statistically significant GAP activity is detected.

To test their possible RGS-Gα coupling systematically, we used a cell-based system that provides a cellular environment to study the action of RGS in the context of GPCR signaling. This assay monitors RGS-induced acceleration of G protein deactivation by real-time bioluminescence resonance energy transfer (BRET) strategy tracking the kinetics of heterotrimer re-association upon antagonizing GPCR, a reaction catalyzed by RGS proteins physiologically (Figure 1A). The key features of the assay include a “bystander” approach that allows the use of unmodified Gα subunits (Figure 1B) and full-length RGS proteins (Figure 1C).

**Figure S1:**
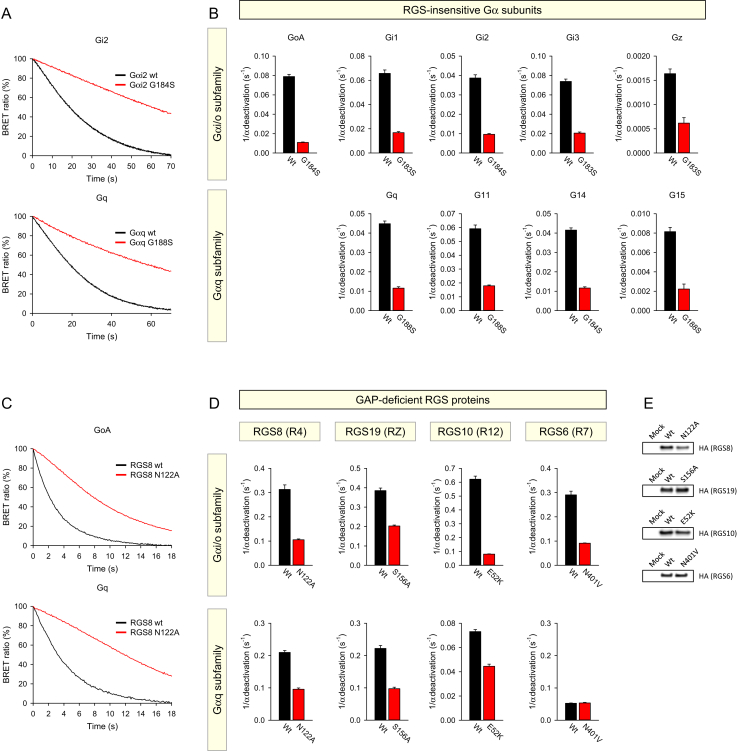
The Effect of Introducing RGS-Insensitive and GAP-Deficient Mutations on the Deactivation Rates, Related to Figure 1 (**A**) The time course of deactivation of wild-type Gα subunits and RGS-insensitive mutants. Each trace represents the mean of the responses measured in three independent experiments. (**B**) Deactivation rate constants of Gα WT and RGS-insensitive mutants. Data are represented as mean ± SEM (n = 3 independent experiments). (**C**) The time course of deactivation of Gα_oA_ and Gα_q_ with RGS8 WT or N122A mutant. Each trace represents the mean of the responses measured in three independent experiments. (**D**) Deactivation rate constants of Gα_oA_ and Gα_q_ with RGS WT or GAP-deficient mutants. Data are represented as mean ± SEM (n = 3 independent experiments). (**E**) Western blot analysis of 3xHA-RGS proteins were performed.

**Figure S2:**
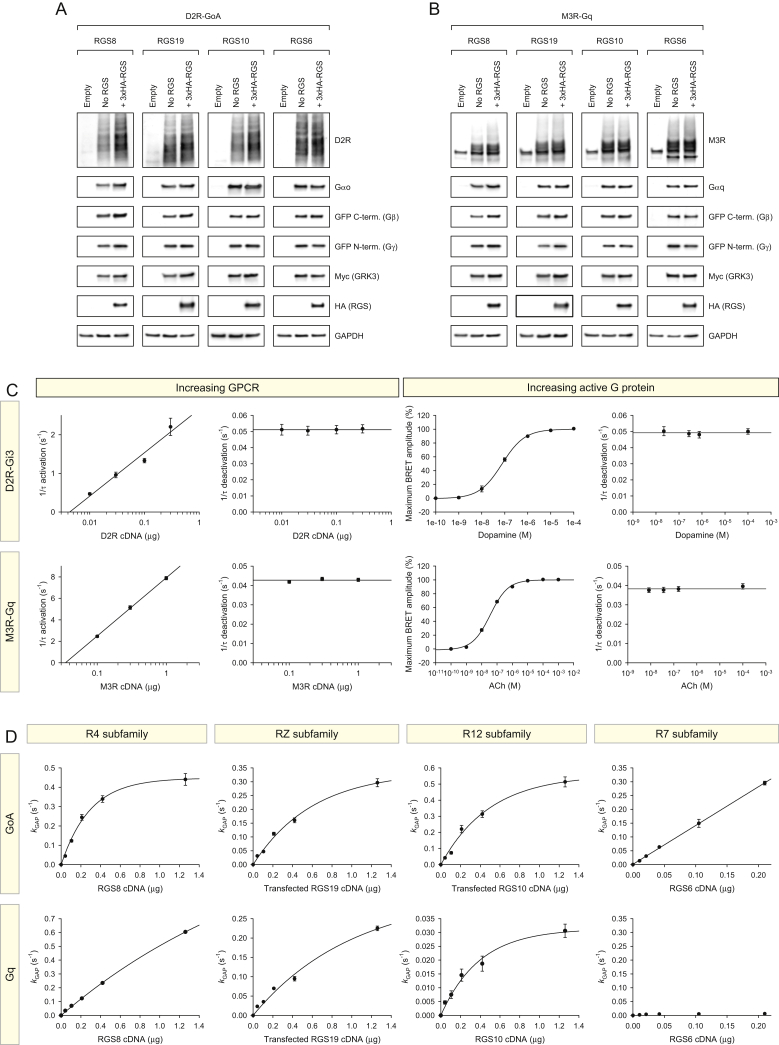
Effects of the Expression Levels of GPCR Signaling Molecules and RGS Proteins on G Protein Deactivation Rates, Related to Figure 1 (**A and B**) Expression levels of GPCR signaling molecules and RGS proteins were examined with western blotting. Overexpression of RGS proteins does not change the expression levels of GPCRs, G proteins, and sensors. (**C**) Effects of increasing GPCR on activation and deactivation rates of G proteins (left). Increasing amount of GPCR cDNA for transient transfection increased G protein activation rates but did not alter G protein deactivation rates. Effects of increasing active G proteins on deactivation rates of G proteins (right). Increasing concentration of agonist produced more active G protein but maintain consistent G protein deactivation rates. (**D**) Effects of increasing RGS on G protein deactivation rates. Increasing amount of RGS cDNA for transient transfection increased deactivation rates.

**Figure S3:**
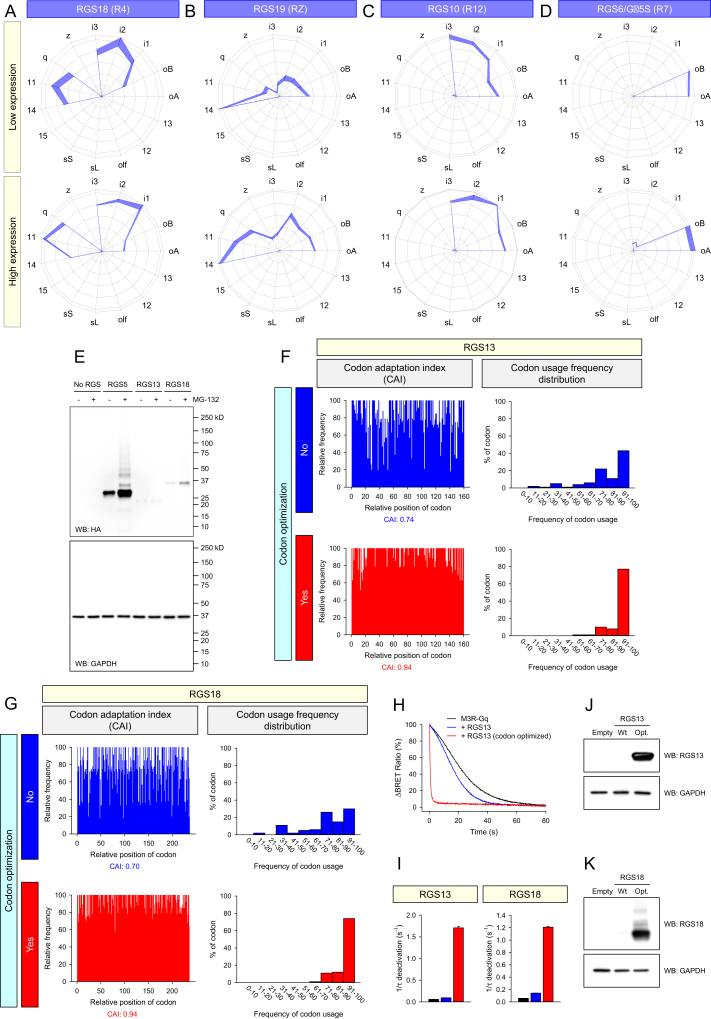
Effect of RGS Expression Level on Gα Selectivity, Related to Figure 1 (**A-D**) Gα-selectivity fingerprints (*k*_GAP_) of RGS18 (**A**), RGS19 (**B**), RGS10 (**C**), and RGS6 (**D**) with low or high expression levels. (**A**) GAP activity of RGS18 before and after codon optimization was compared. High expression condition had 14-fold higher *k*_GAP_ activity relative to low expression (see I). (**B**) HEK293T/17 cells were transfected with 0.42 μg or 1.3 μg of RGS19 for low or high expression, respectively. (**C**) HEK293T/17 cells were transfected with 0.21 μg or 1.3 μg of RGS10 for low or high expression, respectively. (**D**) HEK293T/17 cells were transfected with 0.11 μg or 0.21 μg of RGS6 for low or high expression, respectively, with consistent amount of Gβ5S for both conditions (0.21 μg). The GAP activity on 15 different G proteins was normalized to the largest value to obtain relative *k*_GAP_. The thickness of the lines connecting each data point represents the SEM of three independent experiments. The relative values are plotted on a linear scale. (**E-K**) Optimizing the expression of RGS13 and RGS18. (**E**) Effects of protease inhibitor (MG-132) were examined by western blotting. Cells were treated with 1 μM MG-132 for 4 hours prior to lysing the cells. (**F**) and (**G**) Codon optimization of RGS13 and RGS18. Codon adaptation index (CAI) and codon usage frequency distribution before (blue) and after (red) codon optimization are shown. (**H**) and (**I**) The activity of RGS13 and RGS18 before and after codon optimization. Each trace represents the mean of the responses measured in three wells (**H**). Data are represented as mean ± SEM (n = 3 wells) (**I**). (**J**) and (**K**) Western blot analysis was performed to examine the expression levels of RGS13 and RGS18 with specific antibodies. Western blotting with anti-GAPDH antibody was performed as a loading control.

Using a set of GPCRs with varying Gα selectivity, we recorded the deactivation kinetics of 15 Gα subunits (omitting sensory Gα_t1_, Gα_t2_, and Gα_gust_, but including the two common splice variants of Gα_s_ and Gα_o_) in the absence of exogenous RGS proteins. A combination of intrinsic differences in Gα properties and the action of endogenous RGS proteins in HEK293T/17 cells yielded characteristic baseline deactivation rates (Figures 1D and 1E). Using a previously established approach (Masuho et al., 2013), we ensured that the deactivation kinetics were rate limited by the Gα GTPase activity. Disruption of RGS-Gα interactions by RGS-insensitive (DiBello et al., 1998; Lan et al., 1998) or GAP-deficient mutations (Druey and Kehrl, 1997; Srinivasa et al., 1998) substantially prolonged response recovery (Figure S1). These mutations interfere with the conserved interaction of RGS proteins with the switch I region of the Gα subunits. Further controls demonstrated that (1) the exogenous expression of RGS proteins does not alter the expression of signaling molecules and sensors (Figures S2A and S2B), (2) the different expression levels of GPCRs or the different amounts of active G proteins do not change the G protein deactivation rates (Figure S2C), and (3) deactivation rates are directly proportional to the amount of RGS (Figure S2D). These results confirm that RGS action dictates the kinetics of G protein deactivation. Analysis of the deactivation traces for a representative Gα (Gα_oA_) shows the varying impact of different exogenous RGS proteins on the kinetics of Gα termination (Figures 1F and 1G).

To quantify the activity of RGS proteins, the baseline deactivation rates (1/τ) of each Gα were subtracted from the deactivation rates in the presence of exogenous RGS proteins, yielding the *k*_GAP_ parameter (Figure 1H), a widely used metric of RGS catalytic activity (Ross, 2002). Plotting *k*_GAP_ values for each of the Gα substrates provides a profile of relative activity for a given RGS protein. Analysis of the representative members of the RGS subfamilies using this strategy revealed differences in Gα preferences in a fingerprint-like fashion (Figure 1I). These Gα selectivity fingerprints were not affected by differences in the RGS expression levels (Figure S3A–S3D).

### Principles of Gα Regulation by RGS Family

**Figure S4:**
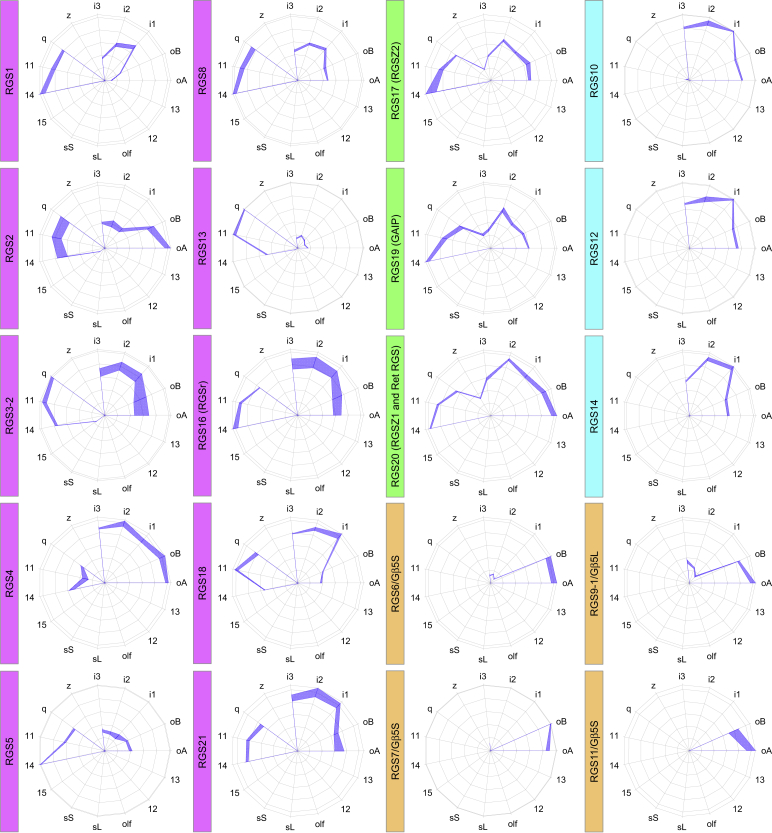
Gα Selectivity of All Canonical RGS Proteins, Related to Figures 1 and 2 Gα-selectivity fingerprints (*k*_GAP_) of all canonical RGS proteins are shown. The GAP activity on 15 different G proteins was normalized to the largest value to obtain relative *k*_GAP_ as shown in Figure 1J. The thickness of the lines connecting each data point represents the SEM of three independent experiments. The relative values are plotted on a linear scale.

This strategy was applied to measure the activity of all 20 canonical RGS proteins on the deactivation of each of 15 Gα subunits in a total of 300 possible combinations. We optimized RGS expression levels, ensuring at least 3-fold acceleration of the deactivation rate for the preferred Gα substrate to reliably assess even minor coupling. Ιn particularly difficult cases (e.g., RGS13, RGS18), proteasomal blockade and codon optimization strategies were applied to augment RGS expression (Figures S3E–S3K). Given the differences in the expression levels of various RGS proteins, we did not attempt to compare their absolute activities and instead focused on elucidating the relative differences in G protein preferences. Collectively, our results provide a comprehensive Gα selectivity profile for the entire RGS family (Figures 1J and S4; Table S1).

**Figure 2:**
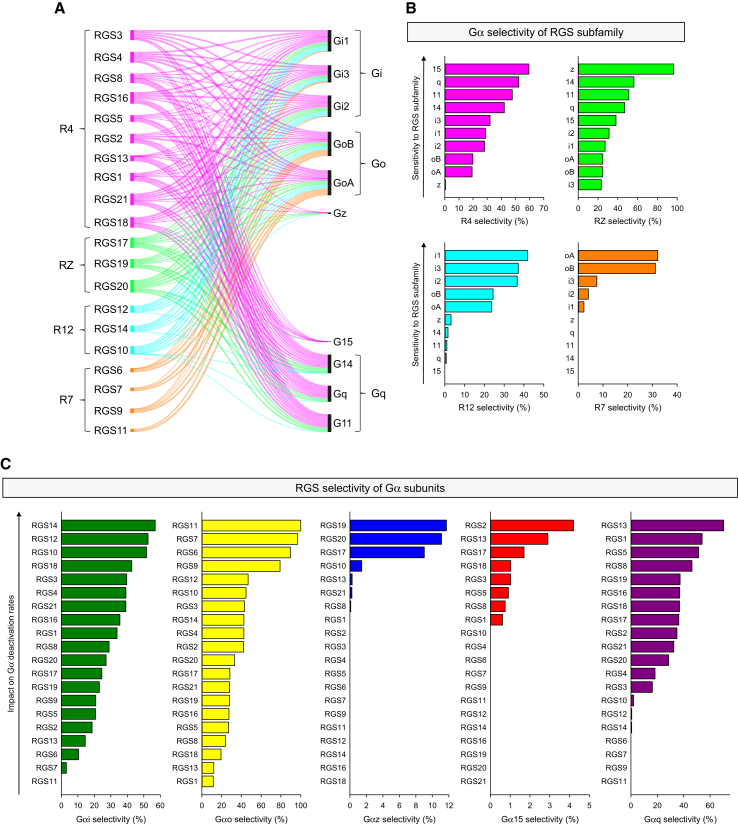
The Complete Network of RGS-Gα Interactions (A) Recognition patterns of Gα by RGS proteins. The width of lines connecting RGS and Gα indicates the strength of GAP activity. Nodes represent total GAP activity of RGS proteins (left side) or on Gα subunits (right side). (B) Gα selectivity of RGS subfamilies obtained by dividing the total GAP activity on each Gα subunit by the number of RGS proteins with statistically significant GAP activity (see Figure S5F). (C) RGS selectivity of Gα subunits obtained by dividing the total GAP activity of an RGS protein on all regulated Gα by the number of Gα subunits (see Figure S5C).

**Figure S5:**
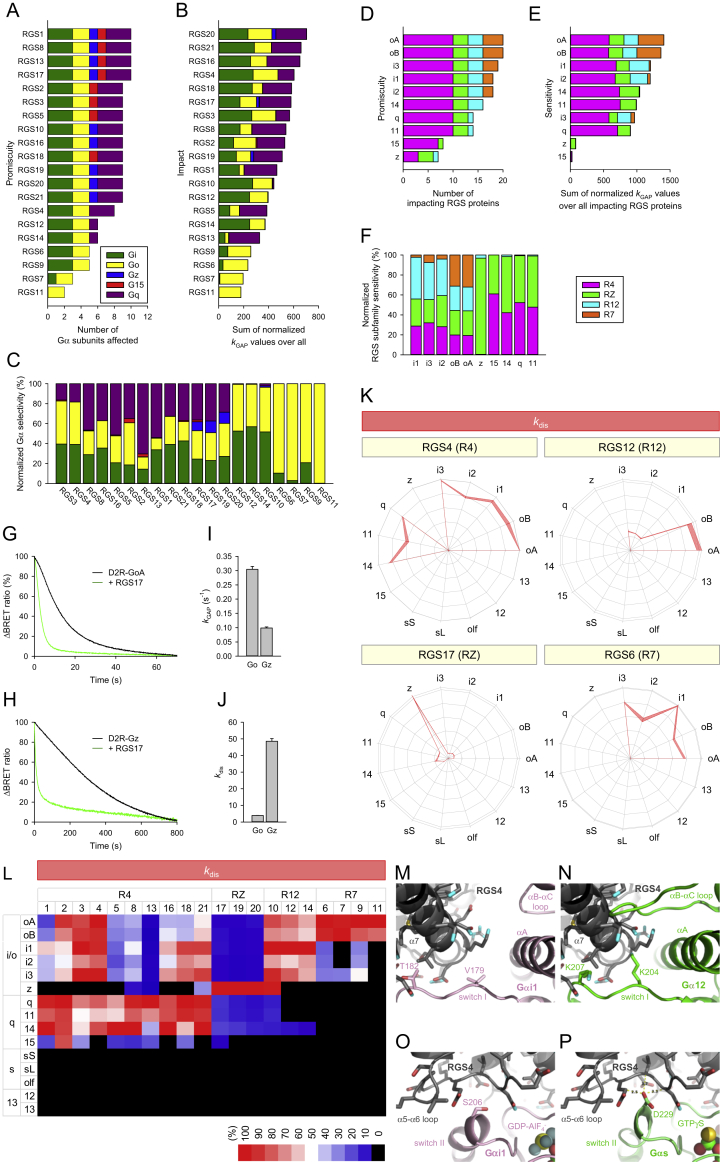
Selectivity of RGS Regulation of Gα Subunits, Related to Figure 2 (**A**) Promiscuity of RGS proteins. The number of Gα subunits affected by each RGS protein was obtained from Figure 2A to determine the range of substrates (promiscuity) for each RGS protein. (**B**) Impact of RGS proteins. The sum of normalized *k*_GAP_ values from Figure 2C was used to quantify the overall impact of each RGS protein. (**C**) Selectivity of RGS proteins. Impact (**B**) was divided by promiscuity (**A**) to obtain normalized Gα selectivity of each RGS protein (**C**). (**D**) Promiscuity of Gα subunits. The number of impacting RGS proteins was obtained from Figure 2B to determine the number of RGS regulating each Gα subunit (promiscuity). (**E**) The sum of normalized *k*_GAP_ values over all impacting RGS proteins was obtained from Figure 2B to determine the sensitivity of Gα subunits to RGS proteins. (**F**) RGS selectivity of Gα subunits. Sensitivity (**E**) was divided by promiscuity (**D**) to obtain normalized RGS subfamily selectivity of each Gα subunits (**F**). (**G-L**) The activity of RZ subfamily on Gαz. (**G**) and (**H**) Effects of RGS17 on the deactivation of GαoA (**G**) and Gαz (**H**). (**I**) and (**J**) The *k*_GAP_ (**I**) and *k*_dis_ (**J**) of RGS17 on Gα_oA_ and G_αz_. Data are represented as mean ± SEM (n = 3 independent experiments). (**K**) Representative Gα selectivity fingerprints of R4, RZ, R12, and R7 subfamilies. The maximum activity (*k*_dis_) from the 15 different G proteins was normalized to the largest value to obtain comparative *k*_dis_ activity and was plotted at corresponding vertices in the wheel diagram. The thickness of the lines connecting each data point represents the SEM of three independent experiments. (**L**) Heatmap of *k*_dis_ of all RGS proteins. (**M-P**) RGS insensitive mechanisms of Gα_12/13_ and Gα_s_. Gα_12/13_ and Gα_s_ have unique surface features that preclude their interaction with RGS proteins. Panels (**M**) and (**O**) depict regions of the RGS4-Gα_i1_ interface from PDB entry 1AGR (RGS4 with gray, Gα_i1_ with pink), whereas panels (**N**) and (**P**) depict Gα_12_ and Gα_s_ (both with green) docked onto Gα_i1_ from the 1AGR structure to highlight their incompatibilities with binding RGS proteins, as represented by RGS4. (**M**) Val179 and Thr182 in switch I of Gα_i1_ and a short αB-αC loop in the helical domain is replaced by Lys204, Lys207, and an extended αB-αC loop, respectively, in Gα_12_ (**N**). These features are conserved in the Gα_12/13_ subfamily and would lead to profound steric collisions with the backbone of a bound RGS domain. (**O**) Ser206 in switch II of Gα_i1_ is replaced by Asp229 in Gα_s_ (**P**) which would introduce van der Waals collisions (dashed lines with numbers corresponding to distances in Å) as well as charge repulsion with an adjacent carboxylate in the α5-α6 loop of RGS4. The Gα_s_-D229S mutation confers the ability of RGS4 and RGS16 to bind Gα_s_, and the ability of RGS16 to accelerate GTP hydrolysis on Gα_s_.

Analysis of the RGS-Gα interaction network provided several key insights. We found that RGS proteins vary markedly in the breadth of their selectivity, with some members (e.g., RGS1) regulating all G_i/o_- and G_q_-type proteins, whereas others (e.g., RGS11) regulated only one Gα type, Gα_o_ (Figures 2A, 2B, and S5A–S5C). The R4 and RZ subfamilies regulated the broadest range of Gα substrates (Figures 2A, 2B, and S5A–S5C). Collectively, R4 and RZ members regulated all Gα_q_ and Gα_i/o_ types with a spectrum of biases (Figures 1J, 2A, and S4). For example, RGS3 and RGS4 preferred the Gα_i/o_ over the Gα_q_, whereas RGS5 and RGS13 selected Gα_q_ over Gα_i/o_. No RGS protein was shown to be specific for the Gα_q_ subfamily. The narrowest selectivity was observed for the R7 subfamily, the members of which regulated Gα_i/o_ proteins exclusively (but not Gα_z_) with prominent selectivity for Gα_o_.

This analysis revealed that Gα subunits vary substantially in their sensitivity to RGS regulation (Figures 2C and S5E). For example, we found Gα_o_ to be the most indiscriminate Gα in that it was regulated by all of the canonical RGS proteins, whereas Gα_z_ could be deactivated only by a limited number of RGS proteins (Figures 2C, S5D, and S5F). We also noticed that a relatively slow rate (0.0021 ± 0.0003 s^−1^) of basal GTPase activity of Gα_z_ possibly underestimated the selectivity of its regulation by RGS proteins when assessed by the *k*_GAP_ parameter (Figures S5G–S5I). Accordingly, we calculated a discrimination index (*k*_dis_) defined by fold increase in the deactivation constant (1/τ) upon the addition of RGS (Figure S5J). Although considering that *k*_dis_ did not change the overall picture of G protein selectivity for most RGS members, it was useful in showing the unique ability of RZ subfamily members to uniquely regulate Gα_z_ (Figures S5K and S5L) amidst their significant activity on virtually all of the other Gα_i/o_ and Gα_q_ proteins based on the *k*_GAP_.

These data also revealed high selectivity in the regulation of the poorly studied Gα_15_. This G protein is activated by a wide range of GPCRs and thus likely contributes to a variety of cellular responses (Offermanns and Simon, 1995). We found that it has a very slow intrinsic deactivation rate (0.0081 ± 0.0006 s^−1^), making RGS regulation paramount for the temporal control of its signaling. Interestingly, Gα_15_ can be deactivated by only a few RGS proteins (Figure S5D), mostly Gα_q_-type-preferring R4 members and an RZ subfamily member, RGS17 (Figures 2C and S5F).

These studies further revealed that no canonical RGS proteins could regulate the deactivation of Gα_s_, Gα_olf_, Gα_12_, or Gα_13_ (Figure 1J). This outcome is perhaps not unexpected. Structural modeling shows that the switch I region of Gα_12/13_ contains Lys-204 instead of a Thr present in all of the other Gα subfamilies in the corresponding position, rendering it incompatible with RGS binding (Figures S5M and S5N). Furthermore, the structure of the αB–C loop in the α-helical domain is also fundamentally different in Gα_12/13_, contributing to the steric occlusion of canonical RGS protein binding (Sprang et al., 2007). Similarly, the presence of Asp229 in Gα_s_, a position conserved as serine in all other Gα subfamilies, renders it incapable of RGS binding in Gα_s_ family members (Natochin and Artemyev, 1998) due to collisions with the α5–α6 loop of RGS proteins (Figures S5O and S5P). The Gα_s_ D229S mutation restores the ability of RGS4 and RGS16 to bind and the ability of RGS16 to accelerate GTP hydrolysis on Gα_s_ (Natochin and Artemyev, 1998).

### RGS-Gα Recognition Patterns Selectively Shape Endogenous Secondary Messenger Signaling

**Figure 3:**
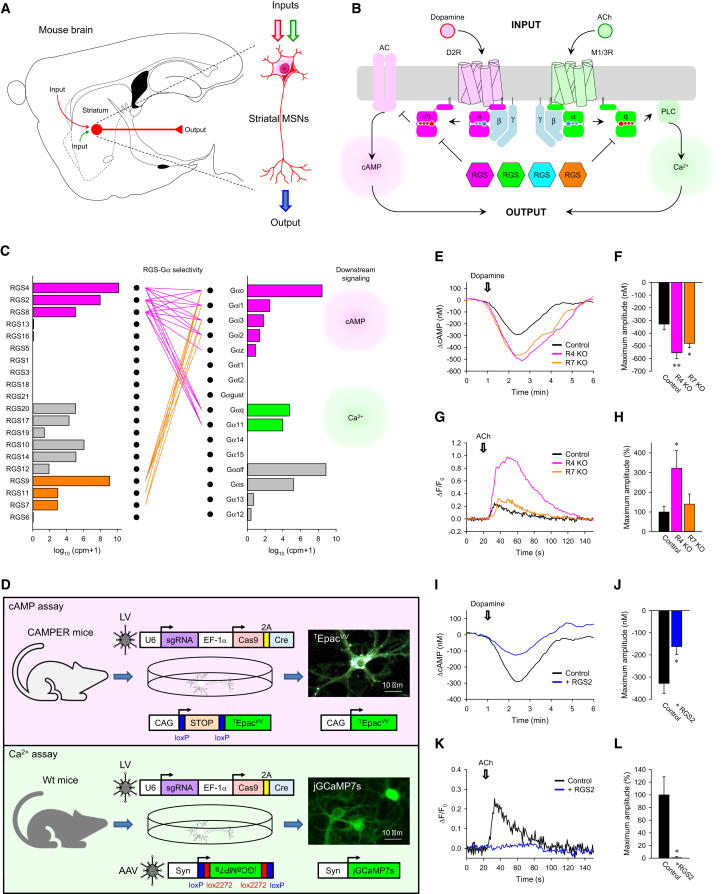
Implications of RGS Selectivity for GPCR Signaling in Striatal Neurons (A) Schematic of the neurotransmitter inputs processing by medium spiny neurons (MSNs) in the striatum. (B) Organization of striatal GPCR signaling cascades and the potential impact of RGS proteins. (C) Analysis of single-cell RNA-seq of MSNs (Gokce et al., 2016) for RGS and Gα expression in alignment with experimentally derived GAP selectivity patterns from Figure 1. (D) Experimental design involving primary striatal neurons from cAMP Encoder Reporter (CAMPER) mice transduced with lentiviral particles containing RGS-targeted single guide RNA (sgRNA) (3 per gene) for CRISPR-Cas9 editing. For cAMP imaging, the CAMPER cAMP sensor was activated by the delivery of Cre recombinase. For Ca^2+^ imaging, neurons were transduced with adeno-associated virus (AAV) particles encoding DIO-jGCaMP7s along with the lentiviral particles for CRISPR-Cas9 editing. (E) Average cAMP response to dopamine (1 μM) in *CAMPER* striatal neurons following CRISPR-Cas9 editing (n = 6–8 neurons). (F) Quantification of maximum cAMP amplitude in (E). (G) Average Ca^2+^ response to acetylcholine (10 μM) in neurons expressing jGCaMP7s following CRISPR gene editing (n = 14–27 neurons). (H) Quantification of maximum Ca^2+^ amplitude from (G). (I) Average cAMP response to dopamine (1 μM) in *CAMPER* striatal neurons following the overexpression of RGS2 (n = 8 neurons). (J) Quantification of maximum cAMP amplitude from (I). (K) Average Ca^2+^ response to acetylcholine (10 μM) in striatal neurons expressing jGCaMP7s following the overexpression of RGS2 (n = 16 neurons). (L) Quantification of maximum Ca^2+^ amplitude from (K). One-way ANOVA followed by Fisher’s least significant difference (LSD) (F and H). Unpaired t test (J) and (L). ^∗^p < 0.05 and ^∗∗^p < 0.01. Data are shown as means ± SEMs from 3–5 independent experiments.

To study how global patterns of RGS-Gα selectivity affect the processing of GPCR signals endogenously, we used striatal medium spiny neurons (MSNs) as a model (Figure 3A). The MSNs were chosen because of their undisputed physiological importance and the critical role of several well-defined GPCRs in processing neuromodulatory inputs to these neurons (Girault, 2012; Xie and Martemyanov, 2011) (Figure 3B). More important, several RGS proteins in the MSNs have been implicated in controlling behavioral responses to GPCR stimulation. The best-documented examples of these are RGS4 (Han et al., 2010; Michaelides et al., 2020), a member of the R4 subfamily, and RGS9 (Traynor et al., 2009), a member of the R7 subfamily.

We surveyed the expression landscape of RGS and Gα proteins by curating the available quantitative RNA sequencing (RNA-seq) data (Gokce et al., 2016). This analysis revealed a significant expression of 12 RGS genes, with RGS4 and RGS9 being the most abundant. Three members of the R4 subfamily (RGS4, RGS2, and RGS8) and 3 members of the R7 subfamilies (RGS9, RGS11, and RGS7) were estimated to be more highly expressed by at least an order of magnitude than other striatal RGS proteins (Figure 3C). Interestingly, our dataset indicates that these RGS subfamilies have distinct patterns of Gα selectivity; the R7 RGS proteins are narrowly tuned for G_i/o_, whereas the R4 RGS members are capable of regulating a broad spectrum of Gα, including both G_i/o_ and G_q_ members (Figures 2B and 2C). Accordingly, transcripts encoding the members (Gα_o_, Gα_i1–3_, Gα_z_, Gα_q_, and Gα_11_) of the Gα_i/o_ and Gα_q_ subfamilies were abundantly expressed by the MSNs (Figure 3C). Thus, we predicted that R4 RGS proteins would have a major influence on the processing of GPCR signals via both G_i/o_ and G_q_ pathways, whereas R7 RGS proteins would selectively affect only Gα_i/o_-mediated signals.

To test this prediction, we used biosensors to monitor the dynamics of second messenger pathway engagement downstream of both G_i/o_ and G_q_ while inactivating RGS proteins by CRISPR-Cas9 editing in the primary cultures of MSNs (Figure 3D). The G_i/o_ activity was assessed by studying its inhibitory influence on cyclic AMP (cAMP) production in response to stimulation of the G_i/o_-coupled dopamine receptor D2 (D2R) by dopamine, whereas G_q_-type activity was monitored by Ca^2+^ transients induced in response to the activation of the muscarinic M1/M3 receptors (M1/3R) by acetylcholine (Figure 3B). Considering the intra-class similarity of RGS-Gα pairing and abundant expression of several members from each RGS class, we chose to simultaneously eliminate all MSN-expressed RGS proteins belonging to the same subfamily by CRISPR-Cas9 editing. The elimination of either the R4 or the R7 subfamily resulted in a significantly enhanced cAMP response, consistent with the role of these RGS members in the deactivation of the G_i/o_ pathway (Figures 3E and 3F). In contrast, the elimination of R4 members but not R7 proteins augmented the Ca^2+^ response, which is in line with their observed Gα selectivity profiles (Figures 3G and 3H).

We next tested the effect of overexpressing individual RGS proteins. We chose to focus on RGS2, an abundantly expressed RGS protein, widely believed to be G_q_ selective based on biochemical measurements but able to regulate G_i/o_ proteins according to our data (Figures 1J and S4). The overexpression of RGS2 had an opposite effect from eliminating RGS proteins and dramatically suppressed the amplitudes of both cAMP and calcium responses (Figures 3I–3L). These observations indicate that the comprehensive RGS-Gα selectivity maps have predictive power in dissecting the logic of GPCR signal processing in an endogenous setting.

### Flexibility of Gα Selectivity Encoded in the RGS Homology Domains

**Figure 4:**
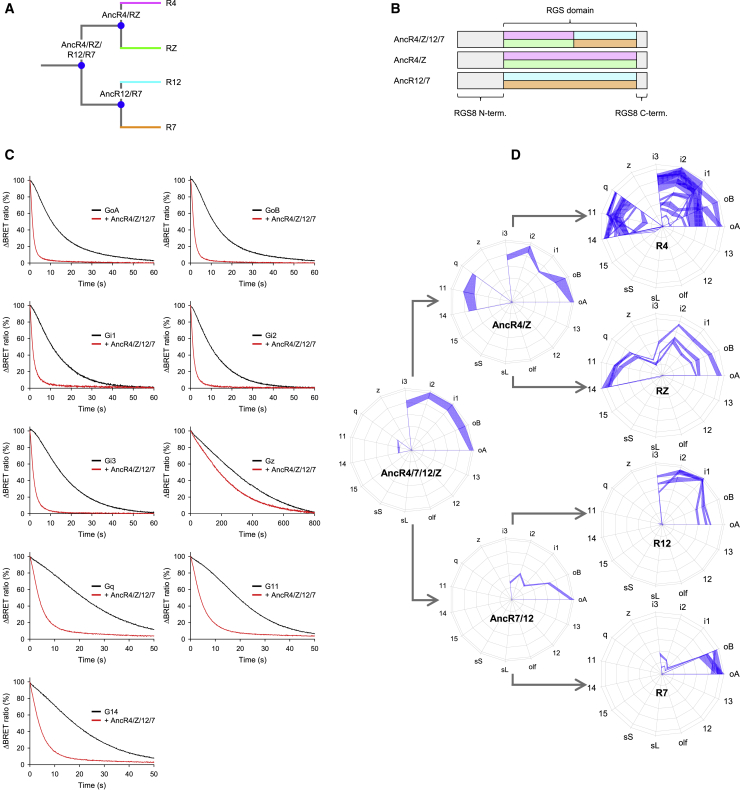
Engineering Gα-Selectivity Fingerprints by Ancestral Reconstitution of RGS Domain Sequences (A) Phylogenetic tree calculated on the basis of multiple sequence alignment of human RGS proteins and a stochastic model of sequence evolution. (B) Schematics of ancestral reconstitution strategy. (C) GAP activity of primordial AncR4/Z/12/7 on Gα subunits with statistically significant activity. (D) Gα selectivity fingerprints of 3 ancestral RGS proteins and extant human RGS proteins.

The analysis presented in this study revealed a wide range of Gα preferences across RGS proteins, which also feature considerable structural diversity (Riddle et al., 2005). This opens questions about the flexibility of recognition patterns across the family and the degree with which Gα selectivity is determined by the RGS domain shared by all RGS proteins. To address these questions in an unbiased way and gain insight into how the selectivity of mammalian RGS subfamilies may have evolved, we performed the reconstitution of ancestral RGS proteins (Figure 4A). We traced the RGS family tree to reconstitute common ancestral RGS domains at three branch points before the diversification into the current four subfamilies and generated a series of chimeric RGS proteins (Figure 4B).

Examination of the Gα selectivity of the primal ancestral RGS protein (AncR4/Z/12/7) revealed that it regulated all Gα subunits that RGS proteins can regulate, except Gα_15_ (Figures 4C and 4D). We next reconstructed two ancestral RGS proteins at the roots of the subfamily divisions (AncR4/Z and AncR12/7). Interestingly, AncR4/Z showed equally strong GAP activity toward Gα_i/o_ and Gα_q_ subfamilies, but not toward Gα_z_ (Figure 4D). Diversification of this precursor RGS subsequently generated various patterns of Gα_i/o_- and Gα_q_ selectivity observed in current R4 and RZ subfamilies. The other ancestral RGS protein, AncR12/7, showed Gα_i/o_ selectivity and was devoid of the ability to regulate the Gα_q_ subfamily. This ancestral RGS gave rise to Gα_i/o_-selective R12 and R7 RGS proteins. These results suggest that Gα selectivity patterns of extant human RGS proteins resulted from a combination of specialization along the Gα_i/o_ versus Gα_q_ axis and *de novo* acquisition of Gα_z_ and Gα_15_ selectivity. This supports a predominantly evolutionary divergence model in which the primordial RGS precursor with balanced activity on different Gα substrates acquired various biases that followed different routes—for example, by suppressing the GAP activity toward the Gα_q_ subfamily in R7 and R12 RGS or re-gaining the activity on Gα_i/o_ subfamily by the R12 RGS. We thus conclude that the sequence composition of the RGS domain has considerable bearing on dictating the evolving Gα preferences of the RGS proteins, strongly suggesting that the major determinants of Gα selectivity are contained within the RGS domain.

### Structural Determinants Governing the Selectivity of Gα Recognition by RGS Proteins

**Figure S6:**
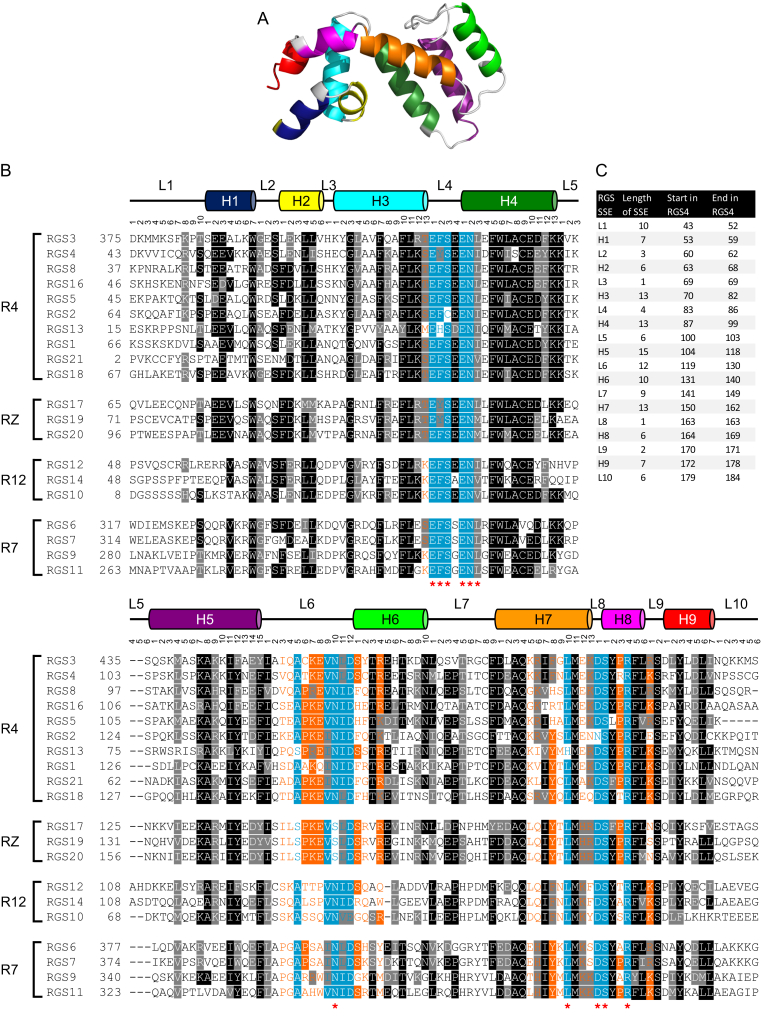
The Common RGS Numbering (CRN) System, Related to Figure 5 (**A**) The structure of the RGS4 RGS domain with color-code for each helix. (**B**) The alignment of all human RGS paralogs with CRN. The common residue numbers are shown on top of the alignment. Directly contacting residues based on the structure of the RGS4/Gα_i1_ complex are highlighted with red asterisks at the bottom of the alignment. The gray indicates the residues with conserved property and black indicate the conserved residues. Of note, there are two insertion/deletion regions in this alignment of the RGS domain. First, there are four amino acid residues in loop 5 in the most of RGS proteins. Instead, there are six amino acids in RGS12 and RGS14, but only three amino acid residues in all R7 RGS members in this structural element. Second, all three R12 RGS proteins are missing an amino acid residue in the H6 region. It is not possible based on existing structural alignments to say where this gap actually occurs, because the H6 region is conformationally heterogeneous in R12 structures and cannot be structurally aligned with other RGS proteins other than to say it has helical character as detected by NMR. The disorder of this region in R12 subfamily members has in fact been proposed to play a role in selecting against the Gα_q_ family due to loss of beneficial interactions with SwIII (Taylor et al., 2016) The conserved and selectivity residues identified by ortholog/paralog analysis (Figure 5C) are highlighted in blue and orange, respectively. The sequence alignments were generated with T-Coffee (http://tcoffee.crg.cat/apps/tcoffee/do:regular) and colored by BoxShade (https://embnet.vital-it.ch/software/BOX_form.html). (**C**) Reference table of the definitions of the secondary structure elements used in the CRN nomenclature. PDB accession number 1AGR is used in panel (**A**).

Elucidation of a Gα-RGS coupling map and demonstration of the crucial role of the RGS domain in determining the pairings prompted the identification of molecular determinants that govern their differential preferences. We compared the sequences of all human RGS domains, aligning them with reference to 20 available high-resolution structures that show the same conserved fold and preservation of key elements, with 9 α-helices and 10 loops (Figure S6A; Data S1). RGS11, RGS13, RGS20, and RGS21 were not included in this analysis because their structures have not been reported. This analysis allowed us to develop a Common RGS Numbering (CRN) system for labeling amino acids relative to their structural position similar to what was previously done for Gα (Flock et al., 2015) and GPCRs (Ballesteros and Weinstein, 1995; Isberg et al., 2015) (Figures S6B and S6C). This system helps to identify the position of every residue with reference to the secondary structure. For instance, RGS4 Asn128, which directly binds to Gα_i1_, is denoted as L6.10, indicating that this residue is the 10th amino acid located in loop 6 of the RGS domain (Figure S6B). It should be noted that this nomenclature cannot be applied to the H6 region in the R12 subfamily because it is structurally distinct from other RGS subfamilies.

**Figure 5:**
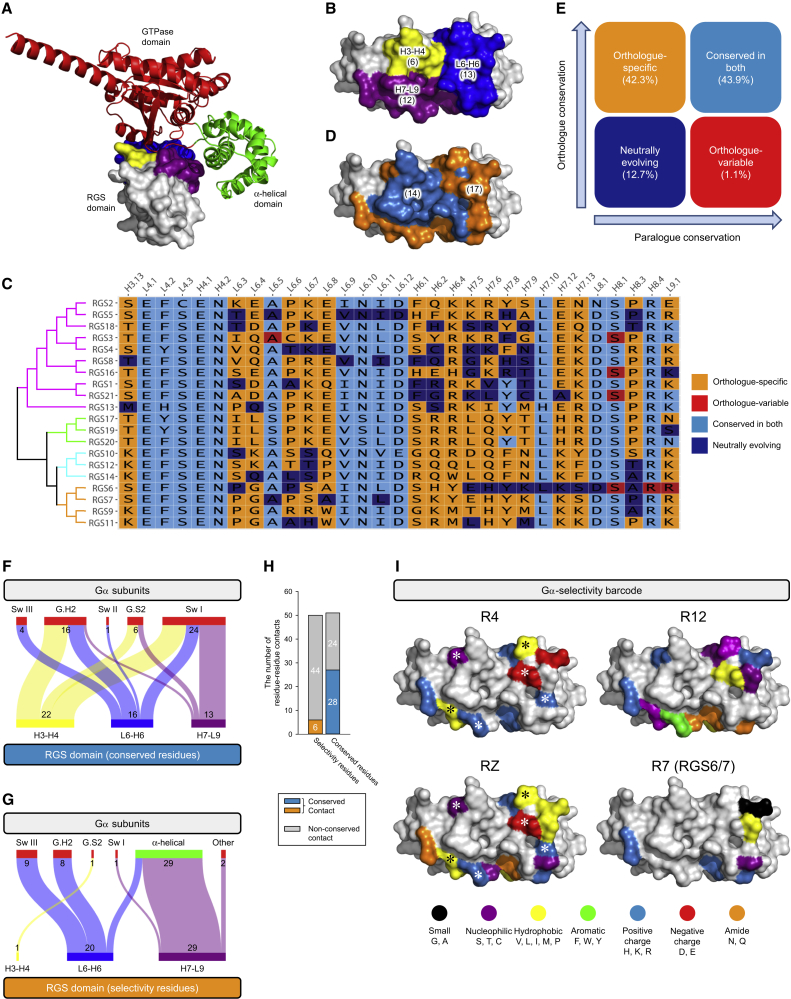
Selectivity Determinants of Gα Recognition by RGS Domain (A and B) Gα-binding surface of RGS domain. GTPase and α-helical domains of Gα subunit are colored red and green, respectively. All of the RGS residues in structural elements within 5 Å from the Gα subunit are colored. The number of residues in each structural element is in parentheses. (C) The selectivity and conserved residues on the Gα-binding surface according to common numbering nomenclature. (D) Mapping the conserved (blue) and selectivity (orange) residues on the Gα-interacting surface of the RGS domain. (E) Quantitative analysis of the ortholog-specific, paralog-specific, neutrally evolving, and conserved residues. (F and G) Interaction network between structural elements in RGS and Gα. The width of the lines indicates the number of non-covalent contacts. The nodes represent the total number of residue-residue contacts for each structural element. Common residue numbering (Flock et al., 2015) is used to indicate the structural elements in the Gα subunit. (H) Quantitative analysis of the number of conserved and non-conserved contacts at the RGS-Gα binding interface. (I) Amino acid properties of selectivity residues with >60% conservation. The asterisks indicate the conserved amino acid residues between R4 and RZ subfamilies. The PDB accession number 1AGR is used in (A), (B), (D), and (I).

We further analyzed eight currently available structures of RGS/Gα complexes and found that all RGS and Gα subunits interact in a very similar manner, with low root mean square deviation (RMSD) in the range of 0.46–1.42 Å. In the RGS domain, there are 11 residues directly contacting Gα that are almost 100% conserved in all structures (Figure S6B). In addition to these contacting positions, we found 20 residues on the RGS protein and 38 amino acids on Gα that contribute to the organization of binding interfaces based on their localization within the 5Å radius of any atom in the interface. On the RGS side, these residues are distributed across 3 structural elements, 2 loops (H3–H4 and L6–H6) and 1 helix (H7–L9) (Figures 5A and 5B). The surface on Gα is more distributed and involves both GTPase and α-helical domains.

To determine which elements most strongly contribute to the selectivity of Gα recognition, we analyzed these 31 RGS residues at the Gα-binding interface across all 20 human RGS paralogs in comparison with their orthologs from 21–65 animal species (Figures 5C and 5D; Data S2). This analysis revealed 14 highly conserved positions across orthologs and paralogs, suggesting that they likely serve as invariable architectural pillars that organize Gα binding and/or GAP activity. These residues included all of the direct Gα-contacting positions found in the RGS4/Gα_i1_ complex (Figure S6B). A minor fraction of the scattered residues was ortholog variable and neutrally evolving (Figures 5C and 5E). The remaining fraction of ortholog-specific residues comprised 17 amino acids. Mapping them on the RGS domain structure showed that they are distributed at the periphery of the Gα-binding surface, surrounding the central positions of the conserved amino acids (Figure 5D), suggesting that they may contribute to Gα selectivity by modulating the interaction. We subsequently refer to these peripheral amino acid residues that are variable among paralogs but conserved within their respective orthologs as Gα selectivity bar codes for RGS proteins.

To identify motifs in the RGS domain that contribute to establishing Gα selectivity, we reconstructed and analyzed the RGS-Gα interaction network at a single amino acid resolution (Figures 5F and 5G). This analysis confirmed that the vast majority of selectivity bar code residues are engaged in non-conserved contacts that vary between different structures of the RGS-Gα complexes (Figure 5H). In contrast, the contacts involving the conserved residues were also predominantly conserved across RGS-Gα structures (Figure 5H). The highest degree of conserved residue-residue contacts is observed for the H3–H4 region with G.H2 and switch I in Gα and for the L7–L9 region with switch I (Figure 5F), indicating its crucial role as a structural backbone for RGS/Gα binding. In contrast, the interaction of the H7–L9 region with the α-helical domain showed the highest number of non-conserved contacts (Figure 5G), suggesting that these domains could significantly contribute to the RGS/Gα selectivity.

To better characterize the organization of the Gα-binding surface, we analyzed properties of the amino acids that form the Gα selectivity bar codes across different RGS subfamilies. This investigation revealed distinct patterns in accordance with the experimentally determined Gα selectivity patterns (Figure 5I). For example, R4 and RZ subfamilies that are dually selective for the G_i/o_ and G_q_ proteins showed a similar distribution of hydrophobic and positively charged residues in the H7–L9 region; hydrophobic and positively and negatively charged residues in L6–H6; and a nucleophilic residue in H3–H4. In contrast, the G_i/o_-selective R12 family exhibited a different pattern featuring nucleophilic, aromatic, and amide residues in the H7–L9 region, and a unique positively charged patch in the L6–H6 lobe surrounded by the nucleophilic cluster. However, another pattern was observed in the narrowly tuned R7 proteins whose L6–H6 region is populated by small amino acids adjacent to the hydrophobic patch and a prominent positive charge in H7–L9. These findings reinforce the idea that the nature of amino acid properties at the selectivity bar code region on the Gα-binding interface of the RGS protein comprises major determinants of Gα recognition selectivity.

### Design Principles for Engineering RGS Protein Selectivity

**Figure 6:**
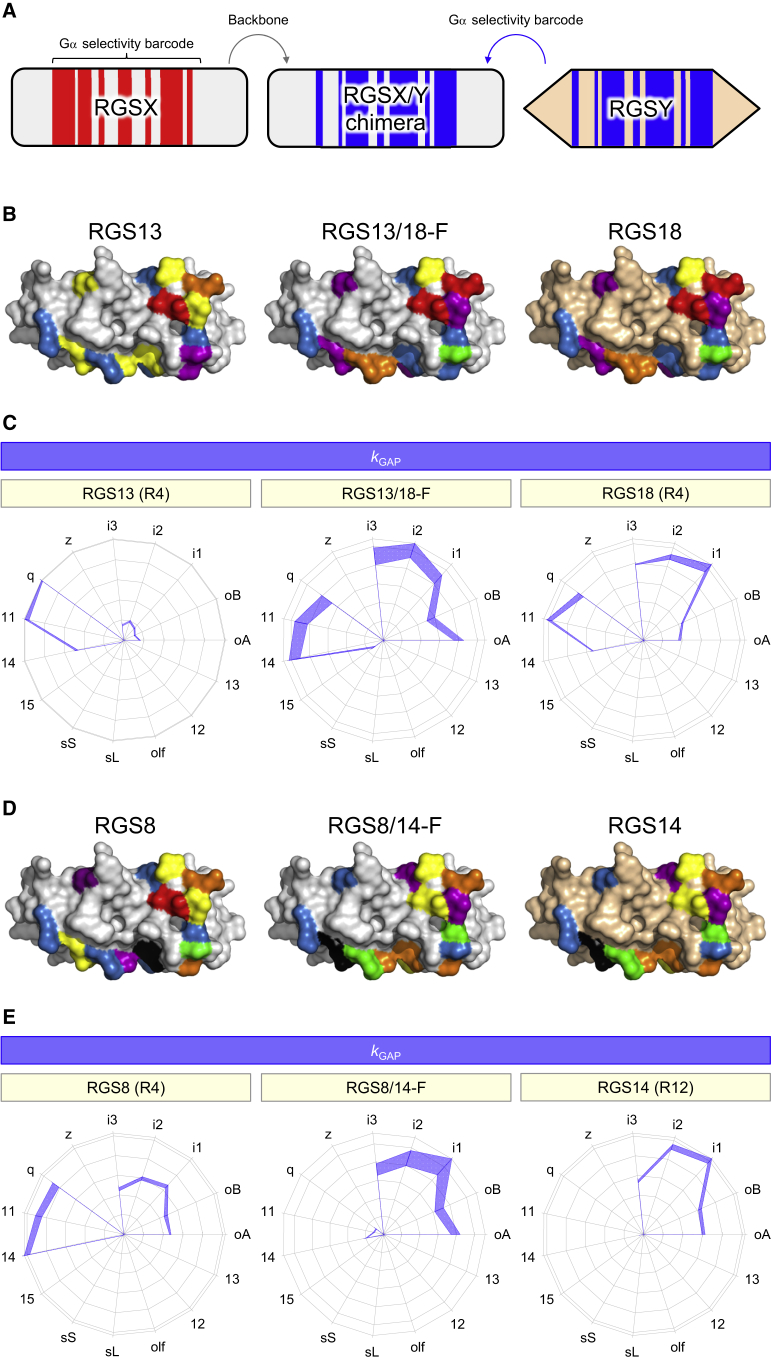
Rewiring Gα Selectivity by Overwriting Gα-Selectivity Bar Codes (A) Scheme for rewiring Gα selectivity. (B) Gα-selectivity bar codes of RGS13 wild type (WT), RGS18 WT, and RGS13/18-F chimera. (C) Gα-selectivity fingerprints of RGS13 WT (left), RGS18 WT (right), and the chimera (center). (D) Gα-selectivity bar codes of RGS8 WT, RGS14 WT, and the RGS8/14-F chimera. (E) Gα-selectivity fingerprints of RGS8 WT (left), RGS14 WT (right), and the RGS8/14-F chimera. Plotted values are means ± SEMs of 3 independent experiments. The PDB accession number 1AGR is used in (B) and (D).

**Figure S7:**
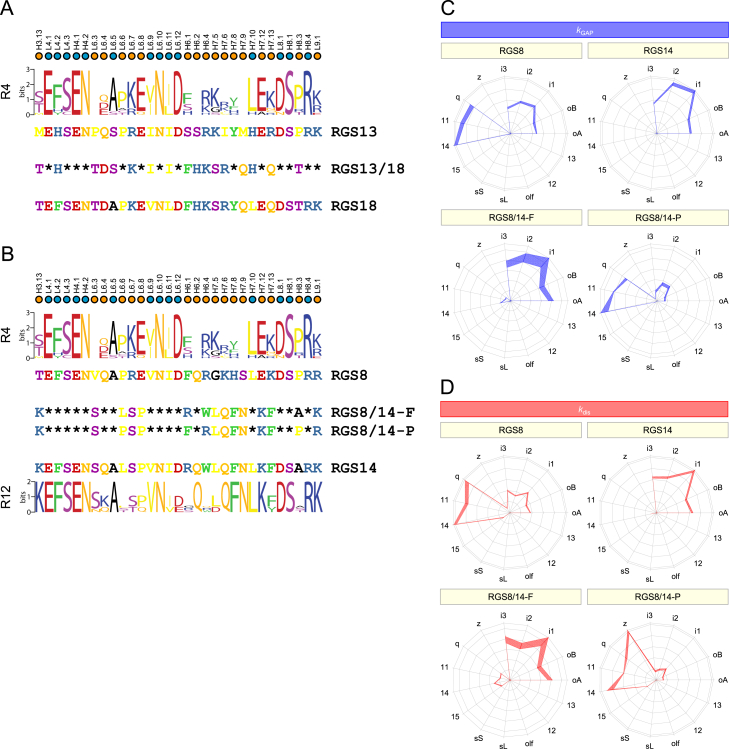
RGS-Gα Selectivity of RGS8, RGS14, and Mutants in *k*_GAP_ and *k*_dis_, Related to Figure 6 (**A**) Sequence pattern of the RGS13, RGS18 and RGS13/18 chimera are shown. Identical amino acid residues between RGS13 and RGS18 were indicated by asterisks. (**B**) Sequence pattern of the R4 and R12 subfamilies, their representative RGS proteins (RGS8 and RGS14), and mutant RGS proteins are shown. Identical amino acid residues between RGS8 and RGS14 were indicated by asterisks. (**C**) and (**D**) The Gα-selectivity fingerprints (*k*_GAP_ (**C**) and *k*_dis_ (**D**)) of RGS8, RGS14, and two mutants are shown. The thickness of the lines connecting each data point represents the SEM of three independent experiments. The relative values are plotted on a linear scale.

The identification of selectivity bar code residues in RGS proteins raises a question about their necessity and sufficiency in setting the selectivity of Gα recognition. This question was addressed experimentally, by transplanting the entire distributed pattern of selectivity residues (Figure 6A). For these experiments, we chose RGS13 and RGS18, which belong to the same R4 subfamily but differ in G protein selectivity (Figure 6C). RGS13 prefers G_q_ members over the G_i/o_ subfamily, whereas RGS18 equally regulates both G_i/o_ and G_q_ proteins. A comparison of their Gα selectivity bar codes indicates that they differ by 12 amino acid residues (Figure S7A). All of the amino acid residues of RGS13 were replaced with the ones from RGS18, resulting in RGS13/18-F chimera (Figure 6B). In agreement with the prediction based on our selectivity bar code model, RGS13/18-F protein exhibited RGS18-like Gα selectivity (Figure 6C).

These experiments were then extended to RGS8 and RGS14, a pair that belongs to different subfamilies and also have markedly different Gα selectivity and composition of Gα selectivity residues (Figures 6D and 6E). We identified 15 different amino acids within the Gα selectivity bar code different between these RGS proteins (Figures 6D and S7B) and transplanted all of these from RGS14 into corresponding positions of RGS8, generating a “full” chimera (RGS8/14-F) (Figure 6D). The RGS8/14-F chimera completely recapitulated the Gα fingerprint of RGS14 without gaining activity on G proteins not regulated by RGS8 or RGS14 (Figure 6E). We further probed whether the change in selectivity could be achieved by mutating fewer bar code residues (i.e., by replacing only nine amino acid residues) (Figures S7B). The resulting “partial” RGS8/14 chimera (RGS8/14-P) had the same Gα_q_ over Gα_i/o_ preference as parental RGS8 (Figure S7C). It thus failed to switch the Gα-selectivity fingerprint from the RGS8 to the RGS14 pattern, indicating that all of the bar code amino acids are required for establishing exact selectivity patterns of Gα-RGS recognition. Curiously, the RGS8/14-P mutant unexpectedly gained activity on Gα_z_ (Figure S7D), indicating that individual residues within the bar code can have an impact on the Gα selectivity of RGS proteins. Overall, these results indicate that identified selectivity bar codes are sufficient in dictating Gα substrate preferences.

### Genomic Landscape of Variability in RGS Selectivity in the Human Population

**Figure 7:**
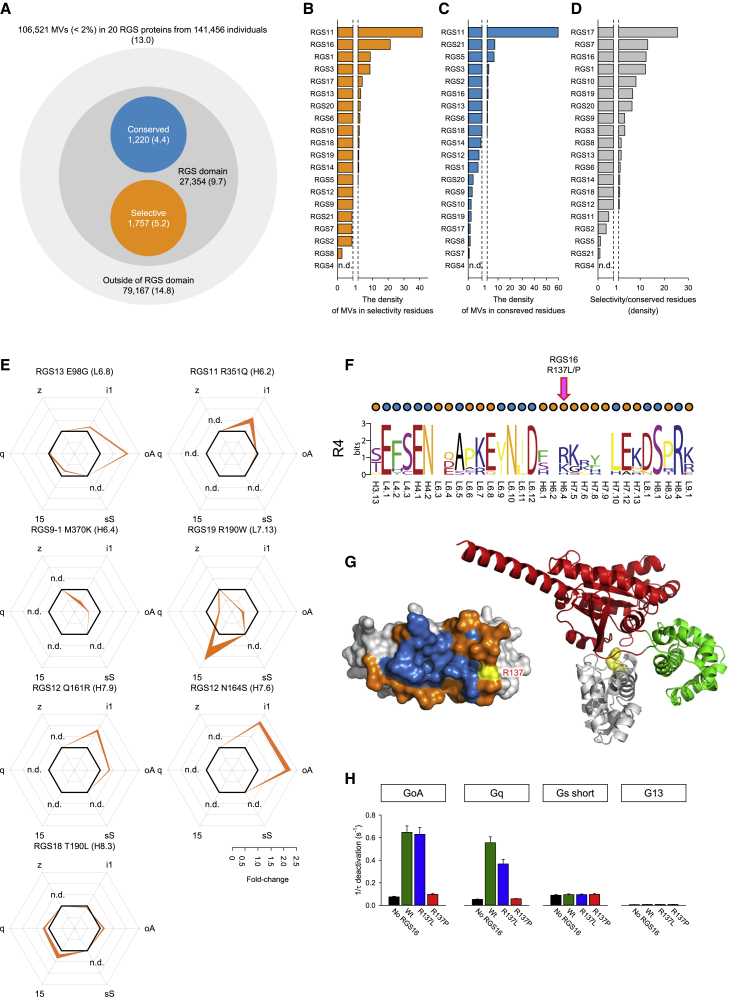
Impacts of Genetic Variation on Gα Selectivity of RGS Proteins (A) The density of MVs as calculated by the number of MVs divided by the number of amino acid residues in each structure. (B and C) The density of MVs in selectivity and conserved residues. If the density is >1, then >1 MV exists in each amino acid residue in the structural element on average. (D) The ratio of the density in selectivity and conserved residues. (E) Functional analysis of MVs on Gα selectivity. The activity of WT RGS proteins is indicated by a black line. The fold change values over the *k*_GAP_ activity of WT RGS proteins are shown. n.d., no significant activity detected. (F) Sequence pattern of R4 subfamily's Gα-binding surface and the position of missense variants in RGS16. (G) The position of RGS16 mutations on the structure of the RGS domain. (H) The effect of the mutations on the function of RGS16. The PDB accession number 1AGR is used in (G). The error bars are SEM values.

To gain insight into how ongoing evolutionary diversification shapes Gα selectivity, we analyzed natural variation in RGS sequences. Prevalence analysis of missense variations (MVs) reported for 2,504 healthy individuals from the 1000 Genomes Project (Auton et al., 2015) revealed that, on average, an individual harbors 5 MVs within the canonical RGS proteins. Examination of the database (Turner et al., 2017) indicated that a *de novo* MV occurs at approximately every 260 newborns, suggesting that RGS proteins are undergoing active evolution. We further analyzed the data on MVs within all of the canonical RGS proteins in 141,456 individuals (Data S3) from the gnomAD database (Karczewski et al., 2020). We found 106,521 rare MVs (minor allele frequency < 2%), with 79,167 MVs on the outside of the RGS domain, 27,354 MVs in the RGS domain, 1,220 MVs in conserved residues, and 1,757 MVs in selectivity residues (Figure 7A). In this analysis, the same variant type is counted multiple times if it occurs in multiple people, illustrating the scale of ongoing evolution (Figures 7A–7D). On average, 13 MVs exist in each amino acid residue of RGS proteins (Figure 7A). This density of MVs (14.8) was the highest outside of the RGS domain. In contrast, functionally important regions exhibited lower densities. The conserved and selectivity residues in RGS11 were the most variable among all of the RGS proteins (Figures 7B and 7C). The ratio of the MV density between selectivity and conserved residues revealed the highest MV frequency in the selectivity residues over the conserved residues in RGS17 (Figure 7D), suggesting likely extensive natural variation of Gα selectivity in RGS17.

To understand the functional implications of the observed variations, we investigated the impact of randomly chosen seven mutations across various positions in the selectivity bar code region of six RGS proteins by testing their activity on the panel of six Gα subunits (Figure 7E). We found that all of the evaluated amino acid changes affected Gα selectivity. Notably, changes at L7.13 in RGS19 (R190W) increased the GAP activity toward Gα_15_, but decreased the activity on Gα_o_, Gα_i1_, and Gα_q_ without any influence on Gα_z_. Alterations in L6.8, H6.2, H7.6, and H7.9 selectively augmented the regulation of Gα_i/o_ without diminishing the activity on other Gα. The balance between Gα_i_ and Gα_o_ regulation can also be affected by these mutations—for example, E98G (L6.8) in RGS13 preferentially increased activity toward Gα_o_ over Gα_i_, while R351Q (H6.2) in RGS11 and N164S (H7.9) in RGS12 augmented Gα_i_ regulation more than Gα_o_. Altering the H6.4 position in RGS9 M370K resulted in a net loss of activity across Gα regulated by this RGS.

Interestingly, variants in RGS proteins are also increasingly viewed as possibly contributing to pathological conditions due to generally disruptive effects (DiGiacomo et al., 2020; Squires et al., 2018). However, the exact mechanisms of functional alterations and implications for Gα selectivity for a vast number of cases remain unexplored. For instance, RGS16 has been recently implicated in insomnia (Hu et al., 2016; Lane et al., 2016), and knockout of this gene in mice disrupts circadian regulation (Doi et al., 2011). The genetic variation (rs1144566) in human RGS16 reported in the genome-wide association study (GWAS) catalog (Buniello et al., 2019) affects selectivity bar code residue H6.4 (Figures 7F and 7G). We experimentally evaluated the functional implication of minor allele variations in H6.4 of RGS16 prevalently occupied by arginine. Our data showed that the R137P mutation nearly completely abrogated the GAP activity of RGS16 for both of its representative preferred substrates, Gα_o_ and Gα_q_, indicating a strong loss of function (Figure 7H). Curiously, the R137L substitution selectively compromised the activity of RGS16 only on Gα_q_ without significant effects on the regulation of Gα_o_. These results indicate that mutations in the selectivity bar code may lead to RGS dysfunction associated not only with the complete loss of function but also with a more subtle alteration in the Gα selectivity.

## Discussion

In this study, we present a nearly complete map of Gα recognition selectivity for all 20 canonical human RGS proteins. The wealth of accumulated evidence in the past 2 decades since their discovery revealed that members of the RGS family exert two distinct effects on the G protein signaling. First, they accelerate G protein deactivation and thus control the duration of signaling. The slow intrinsic GTPase activity of Gα subunits rate limits the termination of the response and does not permit the rapid signaling cycles often demanded by the physiological processes (e.g., in neuronal communication and cardiac activity). By accelerating the Gα GTPase, RGS proteins speed up termination of the response and thereby increase the temporal fidelity of GPCR-initiated signaling. This function is best exemplified by studies on photoreceptors in which the loss of RGS protein in the visual cascade initiated by rhodopsin diminishes the temporal resolution of visual signals, preventing the detection of moving objects (Chen et al., 2000). Second, by deactivating G proteins and/or competing with the effector molecules, RGS proteins interfere with signal propagation, thus taming the extent of signaling (Hepler et al., 1997; Lambert et al., 2010) and allowing adjustment of the signaling volume, depending on the physiological needs. The loss of this RGS function is well noted to sensitize responses causing cellular overreactivity (Lamberts et al., 2013; Neubig, 2015; Xie et al., 2012). From this perspective, RGS proteins could be considered endogenous genetically encoded antagonists of GPCR signaling.

The results of our systematic profiling of RGS substrate preferences prompt reconsideration of the mechanisms involved in cellular signaling diversification. Despite their large numbers, GPCRs can only signal through the same limited number of G proteins that they can activate. Previous studies indicated that signaling diversity is in part dictated by a combination of G proteins activated by individual GPCRs (Inoue et al., 2019; Masuho et al., 2015b). The negative regulation of individual Gα by RGS proteins, if sufficiently selective, would greatly contribute to signaling diversification to allow much more refined signaling characteristics with cellular specificity depending on the available RGS and G proteins. Whereas recent large-scale efforts have provided tremendous system-level insights into the selectivity of G protein activation by GPCRs (Flock et al., 2015; Inoue et al., 2019; Masuho et al., 2015b), the information about the selectivity of RGS has been missing. We fill this gap by establishing Gα selectivity profiles for the entire family of RGS proteins. Based on this information, we propose that RGSs and GPCRs work in synergy to generate diverse cell-type-specific signaling.

Although the experiments presented in this study demonstrate the importance of the bar code residues on the Gα-interacting interface of RGS proteins in dictating Gα preferences, the sufficiency of this residue-residue contact network in dictating precise selectivity patterns across the entire RGS family remains to be tested. It appears quite likely that the secondary network of residues that make contact with the Gα-binding residues on the surface can further adjust and/or reinforce the stringency of Gα recognition. In support of this possibility, members of the R4 subfamily show more diverse functional properties than sequence similarity, suggesting contributions of additional residues within the RGS domain outside of the Gα-interacting surface in shaping Gα selectivity. This is consistent with the results of our ancestral reconstitution experiments, that shuffling wider group of the amino acid residues in the entire RGS domain can also modulate Gα selectivity. Furthermore, elements outside of the RGS domains may further contribute to the Gα recognition preferences of RGS proteins. Such a possibility is suggested by studies on complex multi-modular members of the R7 family, in which interaction partners (Gβ_5_ and R7BP) (Levay et al., 1999; Masuho et al., 2013) and domains (DEP, PGL) (Martemyanov et al., 2003; Skiba et al., 2001) have been shown to regulate Gα recognition. Many RGS genes also produce multiple splice isoforms that alter the structure of RGS proteins by adding or eliminating functionally important motifs without changing the RGS domain (Barker et al., 2001; Chatterjee et al., 2003; Granneman et al., 1998; Saitoh et al., 2002) and may further fine-tune Gα selectivity. Finally, several RGS proteins also interact with GPCRs, G protein effectors, and scaffold proteins (Abramow-Newerly et al., 2006), and this event may further alter Gα specificity. Although these possibilities were not addressed in this study, our experiments with shuffling determinants, mutagenesis, and ancestral reconstitutions all within the RGS domain indicate that these additional mechanisms may contribute to establishing the Gα selectivity but are unlikely to completely overwrite it.

Previous biochemical studies used purified recombinant proteins to examine the preferences of RGS proteins on Gα substrates selected *ad hoc* yielding important information that has served as a reference for RGS-Gα pairing. For example, RGS4 was shown to regulate both Gα_i/o_ and Gα_q_ subfamilies, but not Gα_s_ or Gα_12_ (Berman et al., 1996a; Berman et al., 1996b; Hepler et al., 1997). In contrast, RGS2 was found to have no appreciable GAP activity toward Gα_i/o_ and to be selective for Gα_q_ in both solution GTPase assays and pull-down experiments (Heximer et al., 1997; Kimple et al., 2009). R7 RGS family members were reported to be Gα_o_ selective, with weaker GAP activity on Gα_i_ (Hooks et al., 2003; Posner et al., 1999a; Snow et al., 1998). The selectivity of RGS7 for Gα_o_ over Gα_i_ was observed with the purified RGS domain (Lan et al., 2000), which is consistent with our conclusion that its RGS domain encodes a Gα selectivity bar code. Gα_z_ selectivity of RZ subfamily members RGS17 (RGSZ2), RGS19 (GAIP), and RGS20 (RGSZ1) was also observed (Glick et al., 1998; Wang et al., 1998). Finally, an R12 RGS member, RGS10, has been shown to regulate Gα_o_, Gα_i_, and Gα_z_, but not Gα_s_ (Hunt et al., 1996; Popov et al., 1997). Our investigation confirms many of the previously noted Gα preferences of RGS proteins, while additionally refining them to include G proteins not previously studied. However, in some cases, our results contradict previously documented coupling. One of the notable examples of this is Gα_q_ selectivity of RGS2. Although our investigation shows that RGS2 can indeed regulate several members of the Gα_q_ subfamily, we also find that it exhibits strong activity on the Gα_i/o_ proteins comparable to that on Gα_q_. We think that the discrepancy is largely related to the choice of the assay system. Most of the previous studies used purified RGS and Gα proteins and measured GTP hydrolysis rates using biochemical assays conducted in solution. This approach has limited sensitivity and is devoid of the membrane environment where GPCRs, RGS, and G proteins normally operate under physiological context. In fact, the activity of RGS proteins has been shown to be significantly modulated by the membranes and lipid modification on Gα subunits (Tu et al., 1997). Furthermore, the proteoliposome-based assay was found to yield ∼100-fold higher sensitivity as compared to the solution-based assay (Posner et al., 1999b). RGS2, in particular, was noted to act on Gα_i/o_ in the presence of lipid bilayer (Ingi et al., 1998). Thus, the cellular BRET assay strategy that we chose provides physiologically relevant information on RGS-Gα coupling as it exploits the endogenous environment and appropriate context of RGS action.

One of the key insights provided by this work is the delineation of the determinants involved in RGS-Gα recognition. Establishing principles involved in the selectivity of protein-protein interaction has been a major goal of many investigations (Flock et al., 2017; Nooren and Thornton, 2003). Interaction between RGS and Gα provides an excellent model for interrogation of the underlying principles with possible general implications. Both protein families are well represented by numerous members with clearly defined orthologs and paralogs, and conservation of the structural organization (Baltoumas et al., 2013; Tesmer, 2009). Thus, the experimental definition of the Gα preferences of all of the RGS proteins naturally prompted dissection of the underlying selectivity determinants. This study was focused on examining the contribution of the Gα-binding surface of the RGS domain. A combination of gene orthology/paralogy analysis with structural mapping identified a set of 17 variable amino acids that surround the core critical for forming direct contacts with the Gα subunits. We found that mutations in these amino acids significantly change the Gα preferences of RGS proteins. Interestingly, transplanting sets of variable amino acids from one RGS protein to another completely overwrites the Gα selectivity of the recipient. These observations support the idea that the selectivity of Gα recognition is, at least in part, encoded by the property of the amino acids that form this bar code region on the surface.

Previous studies explored the role of electrostatic interactions in specifying the selectivity of Gα recognition by several RGS proteins across all of the subfamilies (Asli et al., 2018; Israeli et al., 2019; Kosloff et al., 2011; Salem-Mansour et al., 2018). Collectively, these studies reported 12 amino acid residues in RGS proteins that influence their ability to recognize Gα. Mutation of these residues either alone or in combination (up to 7 simultaneously) was shown to either increase or decrease the GAP activity of RGS proteins on the Gα substrates of choice. These studies examined one Gα substrate at a time, thus making it unclear whether the manipulations resulted in switching relative Gα preferences for a given RGS as opposed to overall gain or loss of substrate recognition. Nevertheless, these studies convincingly demonstrate that changes in electrostatic properties of amino acids at the RGS-Gα interface can alter the efficiency of the Gα recognition. Interestingly, all but two (H4.4 and H5.14) of these residues mapped on the Gα selectivity bar code region identified in this study, supporting the idea that electrostatic interactions play an important role in shaping the selectivity of RGS-Gα recognition. Similarly, mutations in RGS2 at the interface with the α-helical domain of Gα subunit diminished GAP activity on Gα_q_ (Nance et al., 2013). In agreement with a large number of contacts made by the α-helical domain with the RGS domain, our analysis shows that variants mapping to this domain in several RGS proteins (H7.6, H7.9, H8.3) affect their Gα selectivity. Taken together with our observations that even single amino acid substitutions within the selectivity bar code can change the Gα preferences of RGS proteins, these results point to critical determinants of RGS-Gα recognition. Curiously, we found that altering these determinants can generate RGS proteins with novel selectivity profiles not displayed by canonical members of the family (e.g., RGS8/14-P, AncR4/Z/12/7; see Figures 4 and 6). Thus, we believe that the Gα-selectivity determinants identified here may pave the way for the *de novo* creation of RGS proteins with rationally designed G protein selectivity.

Our findings also have implications for pharmacogenomics and understanding disease mechanisms associated with the disruption in RGS-mediated G protein control. We uncovered a significant variation affecting nearly all of the RGS proteins. More importantly, many of these variants occurred in selectivity bar code domains and were found experimentally to affect the Gα selectivity of RGS proteins. These genetic alterations are expected to change the profiles of signaling pathways engaged by the GPCRs, creating a situation that the same drug targeting the same receptor would produce varying effects due to RGS heterogeneity. Such a situation may be cryptic in the population if one only profiles variation within GPCRs (Hauser et al., 2018), but it may still lead to interindividual variability in drug response. Therefore, understanding the impact of RGS proteins and their genetic variability on GPCR signaling is expected to be important for individualizing drug prescriptions in the implementation of precision medicine.

## STAR★Methods

### Key Resources Table

**Table:** 

REAGENT or RESOURCE	SOURCE	IDENTIFIER
**Antibodies**		

Anti-GAPDH antibody	MilliporeSigma	Cat# MAB374; RRID:AB_2107445
Anti-HA tag antibody (clone 3F10)	MilliporeSigma	Cat# 11867423001; RRID:AB_390918
Anti-GFP antibody (clones 7.1 and 13.1)	MilliporeSigma	Cat# 11814460001; RRID:AB_390913
Anti-GFP, N-terminal antibody	MilliporeSigma	Cat# G1544; RRID:AB_439690
Anti-c-myc antibody (clone 9E10)	MilliporeSigma	Cat# 11667149001; RRID:AB_390912
Anti-muscarinic acetylcholine receptor m3 antibody	MilliporeSigma	Cat# AB9018; RRID:AB_2080197
Anti-Gα_o_ antibody	MBL life science	Cat# 551; RRID:AB_591430
Anti-Gα_q_ antibody	Santa Cruz Biotechnology	Cat# sc-392; RRID:AB_631537
Anti-dopamine D2 receptor antibody	Santa Cruz Biotechnology	Cat# sc-9113; RRID:AB_2094973
Anti-RGS13 antibody	Novus Biologicals	Cat# H00006003-B01; RRID:AB_1049627
Anti-RGS18 antibody	Novus Biologicals	Cat# NBP1-92329; RRID:AB_11002698
HRP-conjugated anti-rabbit antibody	Jackson ImmunoResearch	Cat# 211-032-171; RRID:AB_2339149
HRP-conjugated anti-mouse antibody	Jackson ImmunoResearch	Cat# 115-035-174; RRID:AB_2338512
HRP-conjugated anti-rat antibody	Jackson ImmunoResearch	Cat# 112-035-175; RRID:AB_2338140

**Bacterial and Virus Strains**		

pGP-AAV9-syn-FLEX-jGCaMP7s-WPRE	Dana et al., bioRxiv 434589	Addgene Plasmid #104491
One Shot Stbl3 *E. coli*	Thermo Fisher Scientific	Cat# C737303

**Chemicals, Peptides, and Recombinant Proteins**		

Dulbecco’s modified Eagle’s medium	Thermo Fisher Scientific	Cat# 11965-092
Fetal bovine serum	Genesee Scientific	Cat# 25-550
Sodium pyruvate	Thermo Fisher Scientific	Cat# 11360-070
MEM non-essential amino acids	Thermo Fisher Scientific	Cat# 11140-050
Penicillin-streptomycin	Thermo Fisher Scientific	Cat# 15140-122
Matrigel	Corning	Cat# 356230
Lipofectamine LTX and Plus reagent	Thermo Fisher Scientific	Cat# 15338-100
Dulbecco’s phosphate-buffered saline	MilliporeSigma	Cat# D5652
Dopamine hydrochloride	MilliporeSigma	Cat# H8502
Haloperidol	MilliporeSigma	Cat# H1512
SCH 39166 hydrobromide	Tocris	Cat# 2299
Acetylcholine chloride	MilliporeSIgma	Cat# A2661
Atropine monohydrate sulfate	MilliporeSigma	Cat# A0257
Bradykinin	Tocris	Cat# 3004
B-9430	BACHEM	Cat# H-7556
Neurobasal-A Medium	Thermo Fisher Scientific	Cat# 10888-022
GlutaMAX	Thermo Fisher Scientific	Cat# 35050-061
B-27 Supplement	Thermo Fisher Scientific	Cat# 17504-044
Penicillin-Streptomycin	Thermo Fisher Scientific	Cat# 15140-122
DNase I	Thermo Fisher Scientific	Cat# 18047019
Poly-D-lysine hydrobromide	MilliporeSigma	Cat# P6407
Papain	Worthington Biochemical	Cat# LS003126
BsmBI	New England Biolabs	Cat# R0580
T4 PNK	New England Biolabs	Cat# M0201
T4 Ligase	New England Biolabs	Cat# M0202
Lipofectamine 2000	Thermo Fisher Scientific	Cat# 11668019
HBSS 10X	Thermo Fisher Scientific	Cat# 14175095
Dopamine hydrochloride	MilliporeSigma	Cat# H8502
Acetylcholine chloride	Tocris	Cat# 2809
Picrotoxin	Tocris	Cat# 1128
CGP 55845 hydrochloride	Tocris	Cat# 1248
DNQX disodium salt	Tocris	Cat# 2312

**Critical Commercial Assays**		

Nano-Glo Luciferase Assay Substrate (furimazine)	Promega	Cat# N1120

**Deposited Data**		

gnomAD	Karczewski et al., 2020	https://gnomad.broadinstitute.org/
denovo-db	Turner et al., 2017	https://denovo-db.gs.washington.edu/denovo-db/index.jsp
GWAS catalog	Buniello et al., 2019	https://www.ebi.ac.uk/gwas/home
Human proteome map	Kim et al., 2014	https://www.humanproteomemap.org/
OMA database	Altenhoff et al., 2018	https://omabrowser.org/oma/home/
Quantitative RNaseq data related to the expression landscape of RGS and Gα	Gokce et al., 2016	https://www.sciencedirect.com/science/article/pii/S2211124716308130

**Experimental Models: Cell Lines**		

HEK293T/17	ATCC	ATCC: CRL-11268

**Experimental Models: Organisms/Strains**		

Mouse: C57BL/6J	The Jackson Laboratory	JAX: 000664
Mouse: C57BL/6-Gt(ROSA)26Sortm1(CAG-ECFP^∗^/Rapgef3/Venus^∗^)Kama/J	The Jackson Laboratory	JAX: 032205

**Oligonucleotides**		

Table S2	This paper	N/A

**Recombinant DNA**		

Plasmid: M3R	cDNA Resource Center	Cat# MAR0300000
Plasmid: D1R	cDNA Resource Center	Cat# DRD0100000
Plasmid: BDKRB2	cDNA Resource Center	Cat# BDKB200000
Plasmid: Flag-D2R	Dr. Abraham Kovoor	N/A
Plasmid: Gα_oA_	Dr. Hiroshi Itoh	N/A
Plasmid: Gα_oA_ G184S	Dr. Osamu Saitoh	N/A
Plasmid: Gα_oB_	cDNA Resource Center	Cat# GNA0OB0000
Plasmid: Gα_i1_	Dr. Hiroshi Itoh	N/A
Plasmid: Gα_i1_ G183S	This paper	N/A
Plasmid: Gα_i2_	Dr. Hiroshi Itoh	N/A
Plasmid: Gα_i2_ G184S	This paper	N/A
Plasmid: Gα_i3_	Dr. Hiroshi Itoh	N/A
Plasmid: Gα_i3_ G183S	This paper	N/A
Plasmid: Gα_z_	cDNA Resource Center	Cat# GNA0Z00000
Plasmid: Gα_z_ G183S	This paper	N/A
Plasmid: Gα_q_	Dr. Hiroshi Itoh	N/A
Plasmid: Gα_q_ G188S	This paper	N/A
Plasmid: Gα_11_	cDNA Resource Center	Cat# GNA1100000
Plasmid: Gα_11_ G188S	This paper	N/A
Plasmid: Gα_14_	cDNA Resource Center	Cat# GNA1400000
Plasmid: Gα_14_ G184S	This paper	N/A
Plasmid: Gα_15_	cDNA Resource Center	Cat# GNA1500000
Plasmid: Gα_15_ G188S	This paper	N/A
Plasmid: Gα_sS_	Dr. Hiroshi Itoh	N/A
Plasmid: Gα_sL_	cDNA Resource Center	Cat# GNA0SL0000
Plasmid: Gα_olf_	cDNA Resource Center	Cat# GNA0L00000
Plasmid: Gα_12_	cDNA Resource Center	Cat# GNA1200000
Plasmid: Gα_13_	cDNA Resource Center	Cat# GNA1300001
Venus-156-239-Gβ_1_	Hollins et al., 2009	N/A
Venus-1-155-Gγ_2_	Hollins et al., 2009	N/A
masGRK3ct-Nluc-HA	Gulati et al., 2018	N/A
masGRK3ct-Nluc-myc	This paper	N/A
Plasmid: Gβ_5S_	cDNA Resource Center	Cat# GNB0500000
Plasmid: Gβ_5L_	cDNA Resource Center	Cat# GNB05L0000
Plasmid: RGS1	This paper	N/A
Plasmid: RGS2	cDNA Resource Center	Cat# RGS0200000
Plasmid: RGS3-2	cDNA Resource Center	Cat# RGS0300002
Plasmid: RGS4	cDNA Resource Center	Cat# RGS0400000
Plasmid: RGS5	cDNA Resource Center	Cat# RGS0500000
Plasmid: RGS6	cDNA Resource Center	Cat# RGS0600000
Plasmid: RGS6 N401V	This paper	N/A
Plasmid: RGS7	cDNA Resource Center	Cat# RGS0700000
Plasmid: RGS8	cDNA Resource Center	Cat# RGS0800000
Plasmid: RGS8 N122A	This paper	N/A
Plasmid: RGS9-1	This paper	N/A
Plasmid: RGS10	cDNA Resource Center	Cat# RGS1000000
Plasmid: RGS10 E52K	This paper	N/A
Plasmid: RGS11	cDNA Resource Center	Cat# RGS1100002
Plasmid: RGS12	cDNA Resource Center	Cat# RGS1200003
Plasmid: RGS13	cDNA Resource Center	Cat# RGS1300000
Plasmid: RGS13 with codon optimization	This paper	N/A
Plasmid: RGS14	cDNA Resource Center	Cat# RGS1400000
Plasmid: RGS16	cDNA Resource Center	Cat# RGS1600000
Plasmid: RGS17	This paper	N/A
Plasmid: RGS18	cDNA Resource Center	Cat# RGS1800000
Plasmid: RGS18 with codon optimization	This paper	N/A
Plasmid: RGS19	cDNA Resource Center	Cat# RGS1900001
Plasmid: RGS19 S156A	This paper	N/A
Plasmid: RGS20	cDNA Resource Center	Cat# RGS2000002
Plasmid: RGS21	This paper	N/A
Plasmid: AncR4/Z/12/7	This paper	N/A
Plasmid: AncR4/Z	This paper	N/A
Plasmid: AnxR12/7	This paper	N/A
Plasmid: RGS13/18-F	This paper	N/A
Plasmid: RGS13/18-P	This paper	N/A
Plasmid: RGS8/14-F	This paper	N/A
Plasmid: RGS9-1 M370K	This paper	N/A
Plasmid: RGS11 R351Q	This paper	N/A
Plasmid: RGS12 Q161R	This paper	N/A
Plasmid: RGS12 N164S	This paper	N/A
Plasmid: RGS13 E98G	This paper	N/A
Plasmid: RGS18 T190L	This paper	N/A
Plasmid: Flag-Ric-8A	Fenech et al., 2009	N/A
Plasmid: Flag-Ric-8B	Von Dannecker et al., 2006	N/A
PTX-S1	Raveh et al., 2010	N/A
Plasmid: pSECC	Sánchez-Rivera et al., 2014	Addgene Plasmid #60820
Plasmid: pCMV-VSV-G	Stewart et al., 2003	Addgene Plasmid #8454
Plasmid: pMDLg/pRRE	Dull et al., 1998	Addgene Plasmid #12251
Plasmid: pRSV-Rev	Dull et al., 1998	Addgene Plasmid #12253

**Software and Algorithms**		

ImageJ	Schneider et al., 2012	https://imagej.nih.gov/ij/download.html
GraphPad Prism 6	GraphPad Software	https://www.graphpad.com/
SigmaPlot 12.5	SYSTAT Software	https://systatsoftware.com/
PyMol	Schrödinger	https://pymol.org/2/
Clampfit 10.3	Molecular Devices	https://www.moleculardevices.com/products/software/pclamp.html
T-Coffee	Notredame et al., 2000	https://www.ebi.ac.uk/Tools/msa/tcoffee/
BoxShade	ExPASy	https://embnet.vital-it.ch/software/BOX_form.html
jFATCAT-rigid algorithm	Prlic et al., 2010	https://www.rcsb.org/pdb/workbench/workbench.do
FastML	Ashkenazy et al., 2012	http://fastml.tau.ac.il/source.php#download
MSAProbs	Liu et al., 2010	http://msaprobs.sourceforge.net/homepage.htm#latest
COCOMAPS	Vangone et al., 2011	https://www.molnac.unisa.it/BioTools/cocomaps/
EMBOSS Needle	EMBL-EBI	https://www.ebi.ac.uk/Tools/psa/emboss_needle/

### Resource Availability

#### Lead Contact

Further information and requests for reagents should be directed to and will be fulfilled by the Lead Contact, Kirill Martemyanov (kirill@scripps.edu).

#### Materials Availability

Plasmids generated in this study will be distributed upon request without restriction.

#### Data and Code Availability

The published article includes all datasets generated and analyzed during this study.

### Experimental Model and Subject Details

#### Mice

All experimental work involving mice was approved by The Scripps Research Institute’s IACUC committee in accordance with NIH guidelines. Mice were housed under standard conditions in a pathogen-free facility on a 12:12 light:dark hour cycle with continuous access to food and water. Male and female *CAMPER (Gt(ROSA)26Sor*^*tm1(CAG-ECFP∗/Rapgef3/Venus∗)Kama*^ and wild-type *C57/Bl6* mice of both sexes aged from postnatal day 0 to postnatal day 3 were utilized in these studies and were not subjected to any prior experiments.

#### Cultures of clonal cell lines

HEK293T/17 cells were obtained from ATTC (Manassas, VA) and grown in DMEM supplemented with 10% FBS, minimum Eagle’s medium non-essential amino acids, 1mM sodium pyruvate, and antibiotics (100 units/ml penicillin and 100 μg/ml streptomycin) at 37°C in a humidified incubator containing 5% CO_2_.

#### Primary cultures of striatal medium spiny neurons

Primary striatal neurons were cultured similar to previous work (Muntean et al., 2018). The striatum from either wild-type or homozygous *CAMPER* pups were rapidly isolated at age P0 in ice-cold HBSS supplemented with 20% FBS, 4.2 mM NaHCO_3_, and 1 mM HEPES. Striatal tissue was washed in HBSS without FBS prior to digestion at 37°C for 15 minutes in a buffer (pH 7.2) containing 137 mM NaCl, 5 mM KCl, 7 mM Na_2_HPO_4_, 25 mM HEPES, and 0.3 mg/ml papain. Striatal tissue was washed three times with HBSS (20% FBS), three times with HBSS, and three times with growth media (Neurobasal-A containing 2 mM GlutaMAX, 2% B27 Supplement serum-free, and 1% Penicillin-Streptomycin). Striatal tissue was then dissociated through pipetting ∼15 times with a standard P1000 pipette in the presence of DNase I (0.05 U/μL) and plated on poly-D-lysine coated glass coverslips. The cells were maintained in a humidified incubator at 37°C and 5% CO_2_. Half of the growth media was replenished every three days. For Ca^2+^ imaging, neuronal cultures from wild-type mice were incubated for 14-18 days with lentiviral-containing supernatant and AAV9-syn-FLEX-jGCaMP7s-WPRE. For cAMP imaging, neuronal cultures from CAMPER mice were incubated for 14-18 days with lentiviral-containing supernatant. Lipofectamine 2000 was used to transfect RGS2 along with control pSECC (1 μg each/coverslip) in wild-type or *CAMPER* neurons as indicated in the text for overexpression experiments.

### Method Details

#### cDNA constructs

M3 muscarinic acetylcholine receptor (AF498917), dopamine D1 receptor (GenBank: NM_000794 with one silent SNP (A1263G)), bradykinin B2 receptor (GenBank: AY275465), Gα_oB_ (GenBank: AH002708), Gα_z_ (GenBank: J03260), Gα_11_ (GenBank: AF493900), Gα_14_ (GenBank: NM_004297), Gα_15_ (GenBank: AF493904), Gα_s_ long isoform (Gα_sL_) (GenBank: NM_000516), Gα_olf_ (GenBank: AF493893), Gα_12_ (GenBank: NM_007353), Gα_13_ (GenBank: NM_006572), RGS2 (GenBank: AF493926), RGS3-2 (GenBank: NM_001282922), RGS4 (GenBank: AF493928), RGS5 (GenBank: AF493929), RGS6 (GenBank: NM_004296), RGS7 (GenBank: AY587875), RGS8 (GenBank: AF300649), RGS10 (GenBank: AF493934), RGS11 (GenBank: NM_003834), RGS12 (GenBank: NM_198227), RGS13 (GenBank: NM_002927), RGS14 (GenBank: NM_006480), RGS16 (GenBank: AF493937), RGS18 (GenBank: NM_130782), RGS19 (GenBank: NM_005873), RGS20 (GenBank: NM_003702), Gβ_5S_ (GenBank: NM_006578) and Gβ_5L_ (GenBank: NM_016194) in pcDNA3.1(+) were purchased from cDNA Resource Center (https://www.cdna.org). masGRK3ct-Nluc-myc, RGS1 (GenBank: NM_002922), RGS9-1 (GenBank: NM_001165933), codon-optimized RGS13, RGS17 (GenBank: NM_012419), codon-optimized RGS18, RGS21 (GenBank: NM_001039152), AncR4/Z/12/7, AncR4/Z, AncR12/7, RGS13/18-F, RGS13/18-P, RGS8/14-F, RGS9-1 M370K, RGS11 R351Q, RGS12 Q161R, RGS12 N164S, RGS13 E98G, RGS18 T190L, and RGS19 R190W proteins in pcDNA3.1(+) were synthesized by GenScript. Flag-tagged dopamine D2 receptors (GenBank: NM_000795) containing the hemagglutinin signal sequence (KTIIALSYIFCLVFA) at the N terminus was a gift from Dr. Abraham Kovoor. The pCMV5 plasmids encoding rat Gα_oA_, rat Gα_i1_, rat Gα_i2_, rat Gα_i3_, human Gα_q_, and bovine Gα_s_ short isoform (Gα_sS_) were gifts from Dr. Hiroshi Itoh. Rat Gα_oA_ G184S was a gift from Dr. Osamu Saitoh. Venus 156-239-Gβ_1_ (amino acids 156-239 of Venus fused to a GGSGGG linker at the N terminus of Gβ_1_ without the first methionine (GenBank: NM_002074)) and Venus 1-155-Gγ_2_ (amino acids 1-155 of Venus fused to a GGSGGG linker at the N terminus of Gγ_2_ (GenBank: NM_053064)) were gifts from Dr. Nevin A. Lambert (Hollins et al., 2009). Flag-tagged Ric-8A (GenBank: NM_053194) in pcDNA3.1 was a gift from Dr. Jean-Pierre Montmayeur (Fenech et al., 2009). Flag-tagged Ric-8B (GenBank: NM_183172 with one missense mutation (A1586G)) in pcDNA3.1 was a gift from Dr. Bettina Malnic (Von Dannecker et al., 2006). The masGRK3ct-Nluc-HA constructs were constructed by introducing HA tag at the C terminus of masGRK3ct-Nluc reported previously (Gulati et al., 2018; Masuho et al., 2015b). PTX-S1 constructs were reported previously (Raveh et al., 2010). pSECC vector (#60820) (Sánchez-Rivera et al., 2014), pCMV-VSV-G (#8454) (Stewart et al., 2003), pMDLg/pRRE (#12251) (Dull et al., 1998), and pRSV-Rev (#12253) (Dull et al., 1998) were purchased from Addgene. Sequences of oligonucleotides used to construct vectors are provided in Table S2.

#### Antibodies

Anti-GAPDH antibody (MAB374), anti-HA tag antibody (clone 3F10) (11867423001), anti-GFP antibody (clones 7.1 and 13.1) (11814460001), Anti-GFP, N-terminal antibody (G1544), anti-c-*myc* antibody (clone 9E10) (11667149001), and anti-muscarinic acetylcholine receptor m3 antibody (AB9018) were purchased from MilliporeSigma. Anti-Gα_o_ antibody (551) was purchased from MBL life science. Anti-Gα_q_ antibody (sc-392) and anti-D2R antibody (sc-9113) were purchased from Santa Cruz Biotechnology. Anti-RGS13 antibody (H00006003-B01) and anti-RGS18 antibody (NBP1-92329) were purchased from Novus Biologicals. HRP-conjugated anti-rabbit antibody (211-032-171), HRP-conjugated anti-mouse antibody (115-035-174), and HRP-conjugated anti-rat antibody (112-035-175) were purchased from Jackson ImmunoResearch.

#### Transfection

For transfection, cells were seeded into 3.5-cm dishes at a density of 2 × 10^6^ cells/dish. After 2 h, expression constructs (total 5 μg/dish) were transfected into the cells using PLUS (5 μl/dish) and Lipofectamine LTX (6 μl/dish) reagents. The GPCR (dopamine D2 receptor (D2R) (1) for Gi/o, M3 muscarinic acetylcholine receptor (M3R) (1) for Gq, dopamine D1 receptor (D1R) (1) for Gs, and bradykinin B2 receptor (BDKRB2) (1) for G12/13), Gα (Gα_oA_ (2), Gα_oB_ (1), Gα_i1_ (1), Gα_i2_ (2), Gα_i3_ (1.5), Gα_z_ (1.5), Gα_q_ (2), Gα_11_ (2), Gα_14_ (4), Gα_15_ (2), Gα_s_ short (6), Gα_s_ long (4), Gα_olf_ (6), Gα_12_ (3), or Gα_13_ (4)), Venus 156-239-Gβ_1_ (1), Venus 1-155-Gγ_2_ (1), masGRK3ct-Nluc-HA (1) were transfected with different amounts of RGS construct (the number in parentheses indicates the ratio of transfected DNA (ratio 1 = 0.21 μg)). RGS1 (12), RGS2 (12), RGS3-2 (6), RGS4 (12), RGS5 (12), RGS6/Gβ_5S_ (1), RGS7/Gβ_5S_ (2), RGS8 (6), RGS9-1/Gβ_5L_ (2), RGS10 (6), RG11/Gβ_5S_ (6), RGS12 (6), RGS13 (6), RGS14 (6), RGS16 (6), RGS17 (6), RGS18 (12), RGS19 (6), RGS20 (6), and RGS21 (12) were transfected to examine comprehensive G protein selectivity. Gα_14/15_ and Gα_olf_ were transfected with Ric-8A (1) and Ric-8B (1), respectively. A construct carrying catalytic subunit of pertussis toxin PTX-S1 were transfected with Gα_z_, M3R, D1R, or BDKRB2 to inhibit the possible coupling of endogenous Gi/o to GPCRs. An empty vector (pcDNA3.1(+)) was used to normalize the amount of transfected DNA.

#### Cell-based GAP assay

Cellular measurements of BRET between Venus-Gβ_1_γ_2_ and masGRK3ct-Nluc-HA were performed to examine GAP activity of RGS protein in living cells (described in detail in Masuho et al., 2015a, 2015b). Sixteen to twenty-four hr post-transfection, HEK293T/17 cells were washed once with BRET buffer (Dulbecco’s Phosphate-Buffered Saline (PBS) containing 0.5mM MgCl_2_ and 0.1% glucose) and detached by gentle pipetting over the monolayer. Cells were harvested by centrifugation at 500 g for 5 min and resuspended in BRET buffer. Approximately 50,000 to 100,000 cells per well were distributed in 96-well flatbottomed white microplates (Greiner Bio-One). The NanoLuc (Nluc) substrate, furimazine (Hall et al., 2012), were purchased from Promega and used according to the manufacturer’s instruction. BRET measurements were made using a microplate reader (POLARstar Omega; BMG Labtech) equipped with two emission photomultiplier tubes, allowing us to detect two emissions simultaneously with the highest possible resolution of 20 ms per data point. All measurements were performed at room temperature. To activate and then deactivate, the final concentration of 100 μM ligands were used. Specifically, dopamine and haloperidol for D2R, dopamine and SCH39166 for D1R, acetylcholine and atropine for M3R, and bradykinin and B-9430 for BDKRB2 were applied on the transfected cells to control the activity of those GPCRs. The BRET signal is determined by calculating the ratio of the light emitted by the Venus- Gβ_1_γ_2_ (535 nm with a 30 nm band path width) over the light emitted by the masGRK3ct-Nluc-HA (475 nm with a 30 nm band path width). The average baseline value (basal BRET ratio) recorded prior to agonist stimulation was subtracted from the experimental BRET signal values and the resulting difference (ΔBRET ratio) was normalized against the maximal ΔBRET value recorded upon agonist stimulation. The rate constants (1/τ) of the deactivation phases were obtained by fitting a single exponential curve to the traces with Clampfit 10.3. *k*_GAP_ rate constants were determined by subtracting the basal deactivation rate (*k*_app_) from the deactivation rate measured in the presence of exogenous RGS protein. Obtained *k*_GAP_ rate constants were used to quantify GAP activity.

#### Western blotting

For each 3.5-cm dish, transfected cells were lysed in 1 mL of sample buffer (62.5 mM tris-HCl, pH 6.8, 2 M urea, 2% SDS, 5% 2-mercaptoethanol, 10% glycerol, bromophenol blue (0.08 mg/ml)). Western blotting analysis of proteins was performed after samples were resolved by SDS–polyacrylamide gel electrophoresis and transferred onto PVDF membranes. Blots were blocked with 5% skim milk in PBS containing 0.1% Tween 20 (PBST) for 30min at room temperature, which was followed by 90 min incubation with specific antibodies diluted in PBST containing 1% skim milk (anti-D2R antibody (1:1,000), anti-M3R antibody (1,1,000), anti-Gα_o_ antibody (1:1,000), anti-Gα_q_ antibody (1:1,000), anti-GFP antibody (1:1,000), anti-HA antibody (1:1,000), anti-c-*myc* antibody (1:1,000), anti-RGS13 antibody (1:1,000), anti-RGS18 antibody (1:5,000), and anti-GAPDH antibody (1:10,000)). Blots were washed in PBST and incubated for 45 min with a 1:10,000 dilution of secondary antibodies conjugated with horseradish peroxidase (HRP) in PBST containing 1% skim milk. Western blotting was performed with BlotCycler automated western blot processor (Precision Biosystems). Proteins were visualized with Kwik Quant imager (Kindle Biosciences).

#### Lentivirus preparation for CRISPR-Cas9 mediated knockout of RGS proteins

As previously described (Doyle et al., 2019; Muntean et al., 2018), sgRNA sequences targeting RGS proteins were designed with CHOPCHOP (https://chopchop.cbu.uib.no/). According to the design, oligo DNAs were synthesized by Integrated DNA Technologies. The oligo DNAs were treated by T4 polynucleotide kinase and annealed in a thermal cycler. Finally, the oligo DNAs were ligated into the BsmBI site of the pSECC vector with T4 DNA Ligase. Three sgRNA constructs were made for each target gene. The plasmids were purified from Stbl3 *E. coli*. Lentiviruses were generated by Lipofectamine LTX-mediated transfection of HEK293T/17 cells with the packaging vectors, pSECC, pCMV-VSV-G, pMDLg/pRRE, and pRSV-Rev. The supernatant containing the lentiviral particles was collected at 48 hours post-transfection.

#### Live-imaging of cAMP and Ca^2+^ dynamics

Primary neuronal cultures were imaged under a Leica TCS SP8 confocal microscope through a 25x objective lens. Changes in cAMP were recorded from *CAMPER* neurons, as previously described (Doyle et al., 2019; Muntean et al., 2018). Briefly, excitation of mTurquoise FRET donor with a 442 nm diode laser was paired with simultaneous acquisition of XYZ image stacks at 10 s intervals collected through two HyD detectors tuned to 465-505 nm (mTurquoise FRET donor) and 525–600 nm (Venus FRET acceptor). Quantification of fluorescence intensity was performed on neuronal cell bodies using ImageJ (Schneider et al., 2012) to calculate FRET from the donor/acceptor ratio. The FRET ratio was converted to the concentration of cAMP using a dose-response curve to cAMP standards in permeabilized neurons. Segregated dopamine receptor subtype expression in striatal neurons enabled the identification of D2R-expressing neurons according to the directionality of cAMP response to dopamine. Dopamine was added in phasic puffs during continuous perfusion (2 mL/minute) of a pH 7.2 buffer consisting of 1.3 mM CaCl_2_, 0.5 mM MgCl_2_, 0.4 mM MgSO_4_, 0.4 mM KH_2_PO_4_, 4.2 mM NaHCO_3_, 138 mM NaCl, 0.3 mM Na_2_HPO_4_, 5.6 mM D-Glucose, and 20 mM HEPES. Changes in intracellular calcium concentration were recorded from wild-type neurons expressing jGCaMP7s. Excitation was performed with a 488 nm laser, and the acquisition of XYZ image stacks at 1 s intervals was collected through a HyD detector tuned to 494–593 nm. Quantification of fluorescence intensity was performed on neuronal cell bodies using ImageJ. Acetylcholine was added in phasic puffs during continuous perfusion (2 mL/minute) of a pH 7.3 buffer consisting of 2.2 mM CaCl_2_, 1 mM MgCl_2_, 138 mM NaCl, 11 mM D-Glucose, 10 mM HEPES, 50 μM picrotoxin, 300 nM CGP55845, and 10 μM DNQX.

#### Alignment of human RGS paralogs and orthologs

Whole protein sequences of human RGS proteins were downloaded from the UniProt database (https://www.uniprot.org/). The core RGS domain in each of these human RGS proteins was assigned based on HMMER searches conducted on pfam database domain profiles using human RGS proteins. Then the core RGS domains assigned in all of the human RGS paralogs were aligned using MSAProbs (Liu et al., 2010) and this alignment was termed as human RGS domain alignment (HRDA). Animal orthologs of RGS proteins were obtained from the OMA database (https://omabrowser.org/oma/home/) (Altenhoff et al., 2018) and equivalent regions to the core RGS domain of human RGS were only considered for further investigations. We aligned the core RGS domain regions in the animal orthologs with human ones. For each human RGS, i.e., RGS1 to RGS21, we constructed multiple sequence alignments of the given RGS with its corresponding animal orthologs.

#### RGS common numbering scheme

We developed a common RGS numbering scheme (CRN), by integrating consensus secondary structure information of available crystal structures of the RGS domain on to HRDA sequence alignment. This allowed us to uniquely assign an alignment position to a combination three types of information: 1) Secondary structural element *i*.*e*. “H” for helix, “S” for strand and “L” for loop, along with the index of the secondary structural element *i*.*e*. “H1” stands for helix number 1 and “L2” stands for loop number 2, etc. 2) Residue number of the alignment position within the index of the given structural element *i*.*e*. “H1.12” denotes 12th position in helix number 1 or helix H1 or L3.2 denotes 2nd position in loop number 3 or loop L3.

#### Normalized BLOSUM scores

For any given alignment position n in the alignment, amino acid residues at this position for across organisms R_i_, where i = 1 to m, where m is the total number of sequences in the alignment.

$$Normalized\;BLOSUM\;score\;\left({NBS}_{n}\right)=\sum_{i=1..m-1}\sum_{i\neq j,j=2..m}BS_{ij}/mC_{2}$$

Where ${\text{BS}}_{\text{ij}}$ = BLOSUM score (R_i_ → R_j_)/Maximum [BLOSUM score (R_i_ → R_i_) OR BLOSUM score (R_j_ → R_j_)] and “ → “ refers to amino-acid residue substitution

Evaluate mean of all over all the “l” positions in the alignment:

$$MeanNBS\;=\;\sum_{n=1..l}{\text{NBS}}_{n}/1$$

#### Orthology/paralogy analysis

To identify the ortholog specific conserved residues and commonly conserved residues between paralogs of human RGS in the core RGS domain. We developed a strategy, by comparing assigning the CRN to each of the RGS alignments and we then categorized the residue at a given CRN position is: (a) Ortholog-specifically conserved if the normalized BLOSUM score for this CRN is 1.5 times higher in a given RGS alignment than in the equivalent CRN of HRDA alignment position and the given CRN position also displays above average normalized BLOSUM score within the RGS alignment. (b) Paralog-specifically conserved if the normalized BLOSUM score for this CRN in the HRDA alignment is 1.5 times higher than in the equivalent CRN of RGS alignment and the given position displays above average normalized BLOSUM score within the HRDA alignment. (c) Conserved in both if CRN in RGS alignment and the HRDA display comparable normalized BLOSUM scores, i.e., within 1.5 times normalized BLOSUM score of either of them. The given position displays above average normalized BLOSUM score within the HRDA and RGS alignments. (d) Neutrally evolving if the above three conditions were not met. The alignment of RGS domain from orthologs is provided as Data S1 and S2. In the datasets, the residue numbers following the accession OMA database ID and UniProt ID or Ensembl database ID are presented.

#### Reconstitution of recombinant ancestral RGS proteins

The reconstitution of ancestral RGS proteins based on the computational algorithm using FastML was performed (Ashkenazy et al., 2012) on different groups of RGS alignments *i*.*e*., for *e*.*g*., R4, RZ, R12, and all RGS proteins. Ancestral reconstruction methods identify most likely sequences, including indels, in a specific ancestral node in a phylogenetic tree for given multiple sequence alignment.

### Quantification and Statistical Analysis

Multiple t tests with correction for multiple comparison using the Holm–Sidak method was conducted to determine the effect of RGS on the deactivation rates of Gα subunits with GraphPad Prism Ver. 6. Only statistically significant values are plotted. Values represent means ± SEM from three independent experiments each performed with three replicates.

## Supplemental Information

Table S1. k_GAP_ and k_dis_ Values of All Canonical RGS, Related to Figure 1

Table S2. Sequences of Oligonucleotides, Related to Figure 3

Data S1. Sequence Alignment of Human RGS Proteins, Related to Figure 5

Data S2. Sequence Alignment of RGS Orthologs, Related to Figure 5

Data S3. Missense Variations of RGS Proteins in the Human Population, Related to Figure 7
